# Deep sequencing of blood and gut T-cell receptor β-chains reveals gluten-induced immune signatures in celiac disease

**DOI:** 10.1038/s41598-017-18137-9

**Published:** 2017-12-21

**Authors:** Dawit A. Yohannes, Tobias L. Freitag, Andrea de Kauwe, Katri Kaukinen, Kalle Kurppa, Pirjo Wacklin, Markku Mäki, T. Petteri Arstila, Robert P. Anderson, Dario Greco, Päivi Saavalainen

**Affiliations:** 1 Research Programs Unit, Immunobiology, University of Helsinki, Helsinki, Finland; 2 Department of Medical and Clinical Genetics, University of Helsinki, Helsinki, Finland; 3 Department of Bacteriology and Immunology, University of Helsinki, Helsinki, Finland; 4 Department of Internal Medicine, Tampere University Hospital and Faculty of Medicine and Life Sciences, University of Tampere, Tampere, Finland; 5 Center for Child Health Research, University of Tampere and Tampere University Hospital, Tampere, Finland; 6 Finnish Red Cross Blood Transfusion Service, Helsinki, Finland; 7 Walter and Eliza Hall Institute of Medical Research, Melbourne, Australia; 8 Institute of Biotechnology, University of Helsinki, Helsinki, Finland; 9 Present Address: ImmusanT, Inc., Cambridge, MA USA; 10 Faculty of Medicine and Life Sciences, University of Tampere, Tampere, Finland

## Abstract

Celiac disease (CD) patients mount an abnormal immune response to gluten. T-cell receptor (TCR) repertoires directed to some immunodominant gluten peptides have previously been described, but the global immune response to *in vivo* gluten exposure in CD has not been systematically investigated yet. Here, we characterized signatures associated with gluten directed immune activity and identified gluten-induced T-cell clonotypes from total blood and gut TCR repertoires in an unbiased manner using immunosequencing. CD patient total TCR repertoires showed increased overlap and substantially altered TRBV-gene usage in both blood and gut samples, and increased diversity in the gut during gluten exposure. Using differential abundance analysis, we identified gluten-induced clonotypes in each patient that were composed of a large private and an important public component. Hierarchical clustering of public clonotypes associated with dietary gluten exposure identified subsets of highly similar clonotypes, the most proliferative of which showing significant enrichment for the motif ASS[LF]R[SW][TD][DT][TE][QA][YF] in PBMC repertoires. These results show that CD-associated clonotypes can be identified and that common gluten associated immune response features can be characterized *in vivo* from total repertoires, with potential use in disease stratification and monitoring.

## Introduction

Celiac disease (CD) is a complex disorder with an overall estimated prevalence of 1% among people of European ancestry^1^. It is characterized by small intestinal villous atrophy leading to nutrient malabsorption but may manifest with a wide range of gastrointestinal and extra-intestinal symptoms. In patients, cereals containing gluten, in particular wheat, barley and rye, activate gluten-specific immunity leading to disease relapse. Consequently, a strict life-long gluten free diet (GFD) is currently the only available treatment for CD.

The most important genetic determinants for susceptibility to CD are Human Leukocyte Antigen (HLA) alleles. About 90% of CD patients carry HLA-DQA1*05 and -DQB1*02 that together encode HLA-DQ2.5, while the remainder carry HLA-DQA1*0301 and -DQB1*0302 (HLA-DQ8), and/or HLA-DQA1*02 and -DQB1*02 (HLA-DQ2.2). HLA-DQ2.5, DQ2.2 and/or DQ8 are found in about half of the general population and are necessary but not sufficient for developing the disease.

CD4+ T-helper cells specific for gluten epitopes presented by HLA-DQ2.5, HLA-DQ2.2, or HLA-DQ8 are considered central to the pathogenesis of CD^2^. Systemic administration of peptides with immunodominant epitopes for gluten-specific CD4+ T-cells causes digestive symptoms that are typically associated with gluten ingestion^3^. CD4+ T-cells specific for gluten are present in the small intestine^2^ and circulate at increased frequencies six days after gluten reintroduction^4^. Peripheral blood collected after short-term gluten challenge harbours expanded populations of gut-homing, effector memory, CD4+ T-cells specific for gluten. In HLA-DQ2.5+ CD patients, gluten-reactive CD4+ T-cells detected in blood by IFNγ ELISpot after short-term wheat, barley or rye challenge preferentially target immunodominant epitopes in one of three short peptides^5^. Gluten challenge in CD patients also increases the frequencies of CD8+ αβ and γδ T-cells, but their antigen specificities have not been determined^6–8^.

CD4+ effector T-cells in blood specific for the most commonly recognized epitopes, HLA-DQ2.5-glia-α2 and HLA-DQ2.5-glia-ω2, show biased but distinct pairing of T-cell receptor(TCR)α and TCRβ genes: *TRBV7-2* with *TRAV26-1* in T-cells specific for HLA-DQ2.5-glia-α2, and *TRBV4* with *TRAV4* in T cells specific for HLA-DQ2.5-glia-ω2^9–11^. CD4+ T-cells specific for either of these epitopes showed features of antigen driven selection such as convergent recombination and semi-public response^9–11^. The semi-public response suggests a common disease mechanism across patients, since random clonotype sharing between individuals is unlikely, owing to the highly diverse T-cell repertoire in individuals generated via the V(D)J recombination machinery^12–14^. Another subset of gluten reactive CD4+ T-cells specific for HLA-DQ2.5-glia-α1a had a biased usage of *TRBV20-1* or *TRBV29-1* ^15^. Similarly, in HLA-DQ8+ CD patients, HLA-DQ8-restricted gluten reactive T-cells show biased usage of *TRBV9-1* ^16^.

Despite such detailed characterizations of isolated T-cells specific for selected immunodominant gluten peptides, a global view of the TCR repertoire directed to *in vivo* gluten exposure is lacking.

Advances in next generation sequencing (NGS) now provide an opportunity to explore the full T-cell response induced by gluten irrespective of whether gluten or other antigens are targeted. This novel approach could provide extensive detail complementary to what has been learned from gliadin-tetramer based approaches^8–11,17^ and systematic gluten epitope mapping studies^5,18^. In this study, we applied deep TCRB CDR3 sequencing to characterize CD patient immune repertoires during gluten exposure, and to identify gluten reactive T-cell clonotypes in an unbiased manner.

## Results

TCR repertoire data was generated from subjects in three experiments (Table 1 and Supplementary Table S1). We obtained an average of 14694 unique productive nucleotide TCRB clonotypes from 544066 reads for each pre- oral gluten challenge (day 0) and post-challenge (day 6) patient PBMC sample. For the gut biopsy samples, there was an average of 8784 unique productive nucleotide TCRB clonotypes from 1414060 reads per patient sample. Clonotypes from the gut were bigger in size (reads/clone) compared to clonotypes in PBMC repertoires, reflecting differences in the proportion of naive and memory T-cells in the tissues (Supplementary Table S1). For sorted samples with CD4+ IFNg+ cells (0.5–3.5% of gated lymphocytes across all samples), we obtained an average of 739 unique productive clonotypes from 114111 reads per individual.

**Table 1:** Information on the study subjects and the treatment conditions for the experiments.

Subject	Diagnosis	Sex	Age	HLA-DQ status	Current diet	GFD duration	tTG IgA serology*	Gluten challenge	Sample	Experiment
CD005	CD	F	61	DQ2.5/DQ2.5	GFD	1 yr	negative	Wheat 3d	PBMC	1,2
CD006	CD	F	61	DQ2.5/DQ5	GFD	16 yr	negative	Wheat 3d	PBMC	1
CD0011	CD	M	55	DQ2.5/DQ8	GFD	10 yr	negative	Wheat 3d	PBMC	1
CD0039	CD	F	55	DQ2.5/DQ2.2	GFD	2 yr	negative	Wheat 3d	PBMC	1,2
HC014	HC	F	30	DQ2.5/DQ6	GFD	4 wk	negative	Wheat 3d	PBMC	1
HC036	HC	F	52	DQ2.5/DQ6	GFD	4 wk	negative	Wheat 3d	PBMC	1
CD037	CD	F	50	DQ2.5/DQ6	GFD	NA	negative	Wheat 3d	PBMC	2
CD025	CD	F	25	DQ2.5/DQ2.5	GFD	6 yr	negative	Barley 3d	PBMC	2
CD027	CD	F	40	DQ2.5/DQ2.5	GFD	1 yr	negative	Barley 3d	PBMC	2
CD034	CD	F	58	DQ2.5/DQ5	GFD	5 yr	negative	Rye 3d	PBMC	2
CD042	CD	F	65	DQ2.5/DQ5	GFD	27 yr	negative	Rye 3d	PBMC	2
CD044	CD	F	64	DQ2.5/DQ6	GFD	7 yr	negative	Rye 3d	PBMC	2
CD1GB	CD	F	67	DQ2/DQ6	AD/GFD	1 yr	90/neg	NA	Biopsy	3
CD2GB	CD	F	47	DQ2/DQ6	AD/GFD	1 yr	57/neg	NA	Biopsy	3
CD3GB	CD	M	56	DQ2/DQ6	AD /GFD	1 yr	21/neg	NA	Biopsy	3
CD4GB	CD	F	20	DQ2/DQ2	AD /GFD	1 yr	44/neg	NA	Biopsy	3
CD5GB	CD	F	39	DQ2/DQ2	AD /GFD	1 yr	>100/neg	NA	Biopsy	3

*tTG IgA negative: <5 U/ml.

### Gluten-specific CD4+ IFNg+ TCRB repertoires are as diverse as total patient PBMC repertoires while patient gut repertoire diversity is increased during active CD

Significant changes in diversity following antigen exposure indicate uneven shift in repertoires, such as mono-, oligo- or poly- clonal expansion, with possible implications in disease development^19,20^. To evaluate TCR repertoire diversity and avoid possible bias due to differences in sequencing depth, we downsampled all samples to 27077 reads (the size of the second smallest sorted repertoire). For each repertoire, the median of 100 diversity estimates from 100 downsamples was used for analysis.

**Figure 1:**
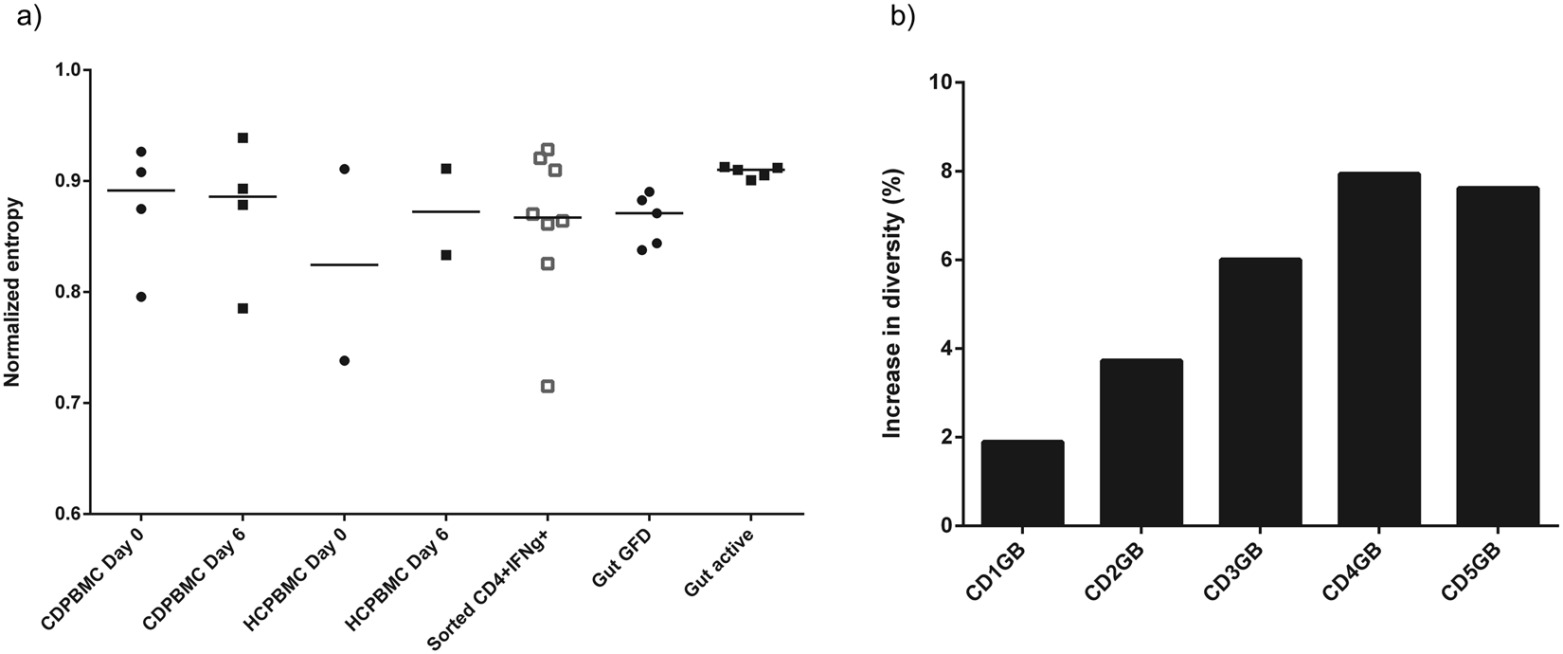
TCR repertoire diversity in gluten exposed CD patients. (**a**) No difference in repertoire diversity is observed between gluten specific CD4+ IFNg+sorted TCRB repertoires and pre- (day0) or post- challenge (day6) CD patient PBMC repertoires. Gut repertoires show interesting increase in diversity (decrease in clonality) during gluten exposure (Wilcoxon paired Signed Rank test, two-tailed, p = 0.06; paired t-test, two-tailed, p = 0.0077. The p-value from the t-test is shown here since the Wilcoxon signed rank test has limitations when applied to small sample size even though it is the main test employed in this study). Repertoire samples were downsampled to 27077 reads 100 times, each time estimating diversity. For each sample, median of the 100 diversity estimates is used for plotting and statistical comparison. Horizontal bars indicate median. (**b**) The diversity increase in active CD gut repertoires ranged from 1.9 to 7.9% (median: 6%) of the treated gut repertoire diversity in patients.

The sorted CD4+ IFNg+ samples did not show any difference in diversity compared to the highly diverse pre- and post- challenge total patient PBMC repertoires (Fig. 1), suggesting high diversity in the sorted gluten-specific TCR repertoires. CD patient post-challenge total PBMC repertoires showed no change in diversity compared to their pre-challenge repertoires. Interestingly, although not statistically significant, all CD patient gut biopsy repertoires showed increased diversity during active disease, with a median increase in diversity of 6% (range: 1.9–7.9%) in biopsies before commencing GFD compared to biopsies collected one year after commencing GFD (Wilcoxon Signed Rank Test, P = 0.06, Fig. 1).

### Gluten exposure induces substantial TRBV gene usage perturbation

**Figure 2:**
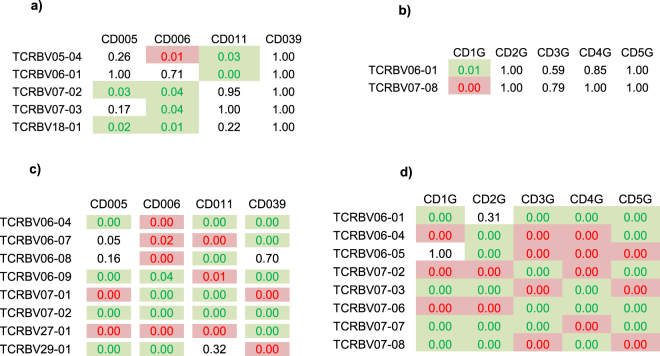
TRBV gene usage is substantially perturbed upon gluten exposure in CD patients. TRBV gene usage in treated versus untreated repertoires is shown for selected gene segments. Heat maps show differentially used TRBV genes in at least one patient (fisher’s exact test, BH adjusted p-values < 0.05), numbers are p-values, green shows odds ratio (OR) > 1 (over-representation) and red OR < 1 (under-representation) during gluten exposure. (**a**) and (**b**) show the result for patient PBMC and gut repertoires respectively when TRBV gene usage is compared at unique clonotypes. (**c**) and (**d**) for patient PBMC and gut repertoires respectively when TRBV gene usage is compared using all reads.

We found no statistically significant difference in mean TRBV gene usage between all post- versus pre- challenge PBMC repertoires, and between all GFD-treated versus active disease gut biopsy repertoires (measured either from unique sequences to analyze overall TRBV gene usage or from total reads to detect expanding/contracting genes) (Supplementary Figure S2). To investigate this further in each individual, we compared TRBV gene usage between the gluten exposed and unexposed repertoires for each subject. When considering unique clonotypes,TRBV5, TRBV6, TRBV7 and TRBV18 family genes were more frequently used in patient total PBMC repertoires after gluten challenge while only TRBV6 showed increased usage in one patient’s biopsy repertoire during active CD (Fig. 2A and B; for all TRBV-gene results see Supplementary Fig. S3). There were no V gene usage differences between unique clonotypes in the sorted and the unsorted repertoires in the two patients CD005 and CD039, from whom we had both sorted and unsorted repertoire data. Importantly, however, substantial TRBV gene usage perturbation was observed when the analysis was performed using total reads (Fig. 2C and D, Supplementary Figure S3). Particularly for the sorted repertoires, strong over-usage of genes was observed in the TRBV18, TRBV19, TRBV29 and TRBV30 families, in addition to those previously reported in TRB6 and TRB7^9–11,15,16,21^ (Supplementary Figure S3E). Overall, TRBV gene usage comparison using total reads (representing all T-cells) detected skewed TRBV gene usage in patients due to antigen induced clonal expansion from the available pool of unique clonotypes.

### Gluten exposure is associated with increased repertoire overlap between CD patients

**Figure 3:**
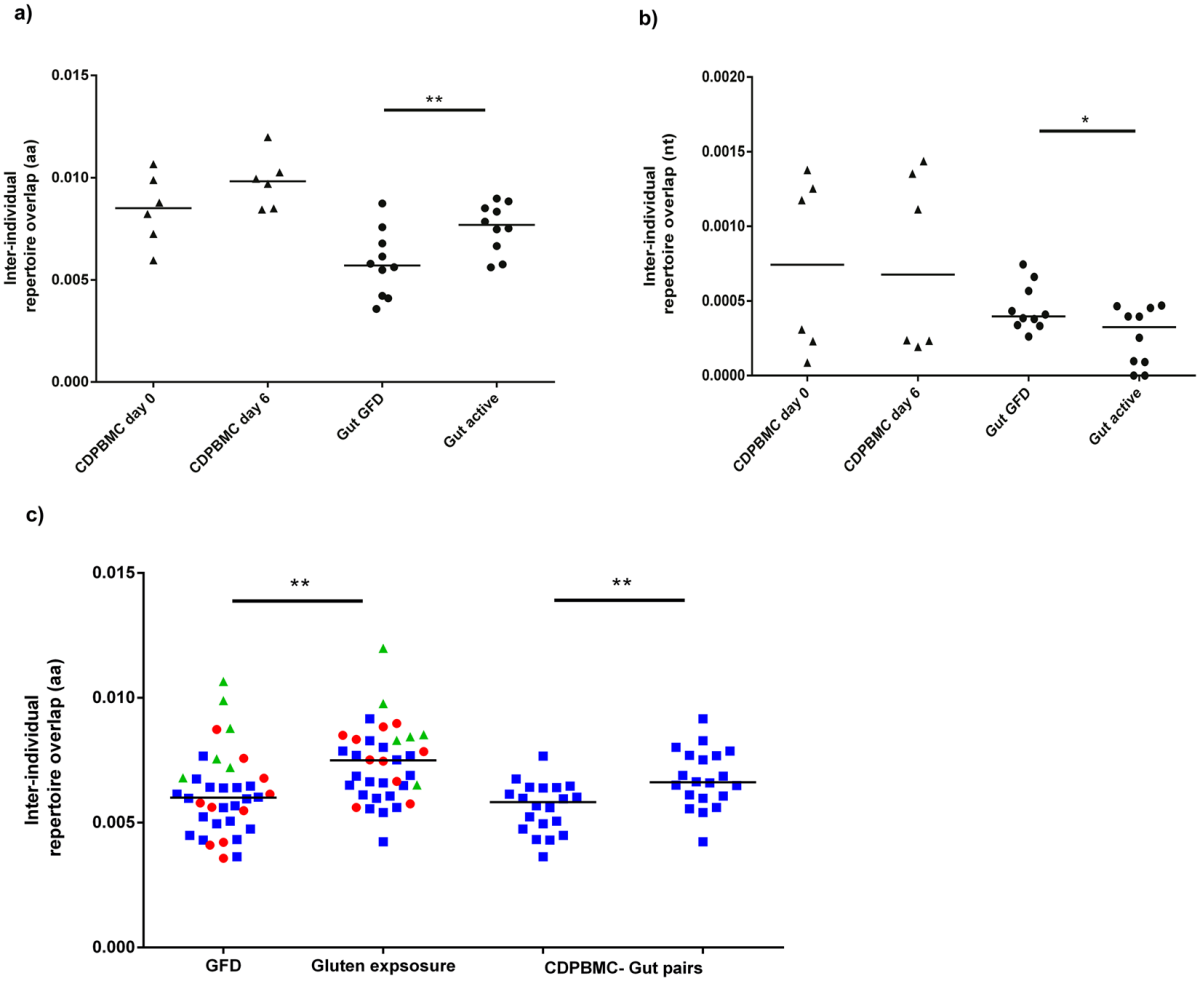
Inter-individual repertoire overlap comparison. (**a**) Increased inter-individual amino acid repertoire overlap is observed in gut biopsy samples during gluten exposure (n = 10 possible pairs at each time point for the 5 CD GUT samples, Wilcoxon paired Signed Rank test, **p = 0.01), while CD patient PBMC repertoires show non-significant increase in overlap. (**b**) No increased inter-individual overlap is observed in the nucleotide repertoires of both PBMC and gut biopsy samples during gluten exposure. (**c**) Inter-individual repertoire overlap for combined data (4 CD PBMC and 5 CD GUT samples, n = 36).The evidence for public component in the response to gluten is stronger when repertoire types are combined for the overlap analysis, showing even higher inter-individual repertoire overlap between unrelated individuals and repertoires drawn from separate tissues (Wilcoxon paired Signed Rank test, **P = 0.0002). PBMC-gut pairs show significantly increased overlap during gluten exposure (Wilcoxon paired Signed Rank test, **P = 0.0064). Horizontal bars indicate median. Patient PBMC pairs are in triangles, gut biopsy pairs are in circles, mixed pbmc-gut pairs are in rectangles.

To assess the effect of gluten exposure on amino acid clonotype sharing between individuals, we compared the inter-individual repertoire overlap between pairs of gluten unexposed repertoires to that of pairs of gluten exposed repertoires. Patient PBMC samples did not show significant difference in overlap after gluten challenge, despite a marginal increase (Fig. 3A). In contrast, there was significantly increased overlap among patient gut biopsy repertoires during active disease compared to biopsies taken after one year GFD (Wilcoxon Signed Rank Test, P = 0.01, Fig. 3A). We also observed increased overlap from the combined comparison of all pairs of patient PBMC and GUT repertoires (Fig. 3C), indicating that the gluten induced public clones in blood are at least partly the same as those residing in the gut even in unrelated patients. These results overall suggest that increased repertoire overlap among patients is a feature of the immune response to gluten. On the other hand, this increasing overlap was not seen when using nucleotide clonotypes for the analysis (Fig. 3B), possibly due to the gluten-induced higher repertoire diversity at the nucleotide level, detectable number of which is likely coding for the same amino acid clonotypes which are shared among patients.

### Differential abundance (DA) analysis identifies gluten-induced and -specific T-cells

**Figure 4:**
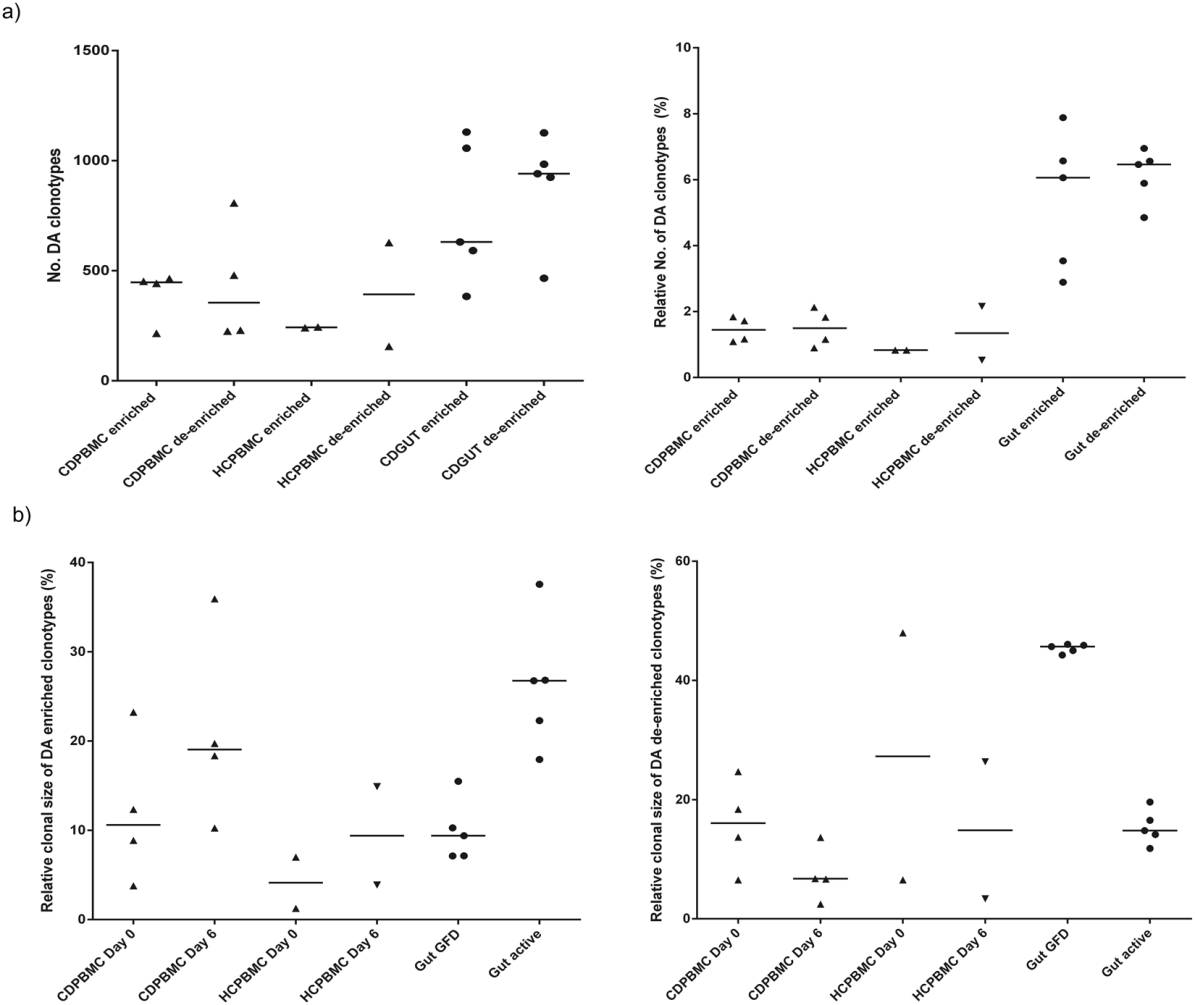
Enriched and de-enriched clonotypes in each subject. Differential abundance analysis between the gluten unexposed and gluten exposed repertoires of each subject was performed. (**a**) Left panel, the analysis determined a median of 448 enriched (i.e., substantially higher frequency) (range: 216–465) and 355 de-enriched (i.e., substantially lower frequency) (range: 226–808) clones following gluten challenge in CD patient PBMC repertoires. In healthy controls, 243 enriched (range: 241–245) and 393 de-enriched (range: 157–628) clonotypes were estimated. For gut repertoires, for which samples were taken one year apart, an average of 631 enriched (range: 384–1130) and 941 de-enriched (range: 466–1127) clonotypes were observed during active disease. Right panel, the number of both enriched and de-enriched unique amino acid clonotypes relative to the total number of all unique amino acid clonotypes observed in the two repertoires of each subject is shown. (**b**) The relative clonal size (relative to total sequencing reads) of the enriched and de-enriched clonotypes, before and after gluten exposure. For both enriched and de-enriched, the combined clonal size of the DA clonotypes in each subject is shown. Horizontal bars indicate median.

We applied the first DA analysis approach (described in the Methods section) to identify DA clonotypes in each individual. For the four patient PBMC repertoires, we found a median of 448 enriched (range: 216–465) and 355 de-enriched (range: 226–808) clones following gluten challenge while the two healthy controls had 243 enriched (range: 241–245) and 393 de-enriched (range: 157–628) clonotypes. For the five gut biopsy repertoires, a median of 631 enriched (range: 384–1130) and 941 de-enriched (range: 466–1127) clonotypes were observed during active disease. These results give a putative estimation of the amount of gluten-induced T-cell clonotypes, which we defined as clonotypes showing significant change in clonal size as a result of gluten exposure regardless of their specificity (Fig. 4).

For the two patients (CD005 and CD039) that had sorted and unsorted repertoires, DA clones identified from their sorted repertoires, compared to their unsorted pre-challenge and post-challenge samples, showed low overlap with those detected from the comparison of their unsorted post-challenge versus pre-challenge repertoires (Supplementary Table S2 and Figure S4). Since DA clonotypes identified from sorted CD4+ IFNg+ repertoires are specific to gluten, the low overlap perhaps indicates that the T-cells specific to the 3 immunodominant gluten peptides among *in vitro* stimulated sorted clones represent only a fraction of all clonotypes enriched after *in vivo* gluten challenge, the latter including both gluten specific and non-specific T-cells. We also found little overlap in gluten-induced DA clones across patients, despite the increased overlap in patient total repertoires during gluten exposure (Fig. 3B and Supplementary Figure S4).

Given the observation that gluten exposure increased inter-individual repertoire overlap, we applied the second DA analysis approach (see Methods section) and evaluated the differential abundance of 11834 public clonotypes that were observed in at least two different individuals (constituting 4.7% of the total 251259 unique amino acid clonotypes available in all samples). This analysis was performed separately for the CDPBMC (n = 4), HCPBMC (n = 2), and CDGUT (n = 5) repertoires (Table 2). From the resulting public DA clones, only two of the public enriched DA clonotypes (CASSLGDTQYF and CASSFSYEQYF) from patient PBMCs overlapped to those detected from healthy PBMCs, and one (CASSLTWDTEAFF) to those detected from patient gut repertoires (Supplementary Figure S4). More than half of the public DA clonotypes were among the recently published 10692 representative public TCRB clonotypes (repPC)^14^, indicating possible clinical utility in maintaining detailed repository of public TCR clonotypes and their specificities. The list of public enriched clonotypes is available in the supplementary dataset 1.

**Table 2:** Differential abundance analysis results on public clonotypes.

				DA clonotypes in:										
	Repertoire type	No. of Clonotypes (RP Pvalue < 0.01)^+^	No. of DA clonotypes (SFS Accuracy > = 0.75)*	Sorted	Total CDPBMC	CDPBMC 0	CDPBMC 6	Total CDGUT	CDGUT GFD	CDGUT active	study (Qiao *et al*.)^11^	study (Han *et al*.)^7^	study (Petersen *et al*.)^15^	repPC (Britanova *et al*.)^14^
**Enriched Public clonotypes**	CDPBMC	70	33	4	33	11	33	17	14	14	3	1	1	21
	HCPBMC	95	95	5	51	26	36	37	26	24	0	0	0	47
	CDGUT	60	30	0	15	8	13	30	15	30	0	1	0	15
**Deriched public clonotypes**	CDPBMC	75	38	3	38	38	16	18	10	17	0	0	0	22
	HCPBMC	102	101	4	56	35	36	46	29	31	0	0	0	53
	CDGUT	45	0	0	0	0	0	0	0	0	0	0	0	0

^+^RankProduct(RP) results from approach two in the DA analysis.

*Sequential forward feature (SFS) selection applied on the RP output resulting in differentially abundant (DA) clonotypes.

**Figure 5:**
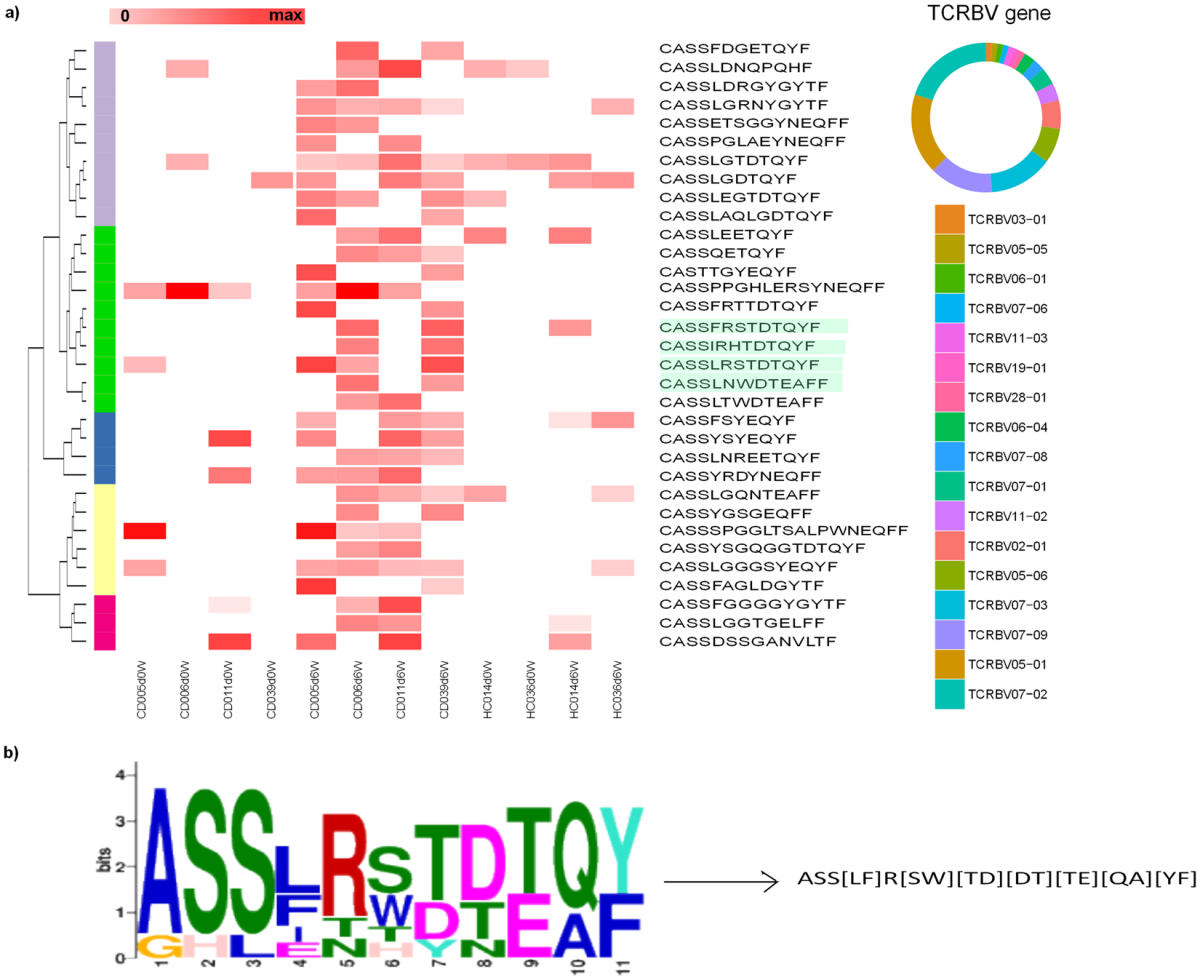
Public differentially enriched CD patient PBMC clonotypes. (**a**) DA clonotypes are enriched in post-challenge CD patient samples (color intensity indicates prevalence level), their prevalence in healthy control pre- and post-challenge samples is also shown for comparison. The donut plot on the right shows the TCRBV genes used by these DA clonotypes in CD patient post-challenge samples, from the least to the most used. Clonotypes were hierarchically clustered using a modified Levenshtein distance that took physicochemical properties into account; the cluster indicated with the green bar has the highest average fold change increase during gluten exposure. (**b**) MEME motif search of the top cluster: significantly over-represented motif shown in sequence logo and regular expression, ASS[LF]R[SW][TD][DT][TE][QA][YF] (E-value = 2.4E-10); Top cluster contains previously known HLA-DQ2 restricted, alpha gliadin epitope specific CD4+ clonotypes with the ASSxRxTDTQY motif (highlighted with light green).

We also investigated the presence of previously reported gluten reactive TCRB CDR3 clonotypes^7,11,15^ in our public DA clonotypes. We found 4 previously reported clonotypes: CASSLRSTDTQYF, CASSFRSTDTQYF, CASSIRHTDTQYF and CASSLNWDTEAFF among enriched clones in patient PBMCs, and CASSLGYEQYF among enriched clones in patient gut repertoires. We did not find any previously known clonotype in the de-enriched clonotypes of patient PBMC repertoires and enriched/de-enriched clones of healthy control PBMC repertoires (Table 2), consistent with the assumed gluten-specificity of these clones. Next, we examined sequence similarity between public DA clonotypes to identify conserved structures. Hierarchical clustering of the 33 enriched CDPBMC clonotypes resulted in five clusters. The top cluster had a significant over-representation of the MEME regular expression motif ASS[LF]R[SW][TD][DT][TE][QA][YF] (Fig. 5). The same analysis for the de-enriched clonotypes resulted in six clusters with the top cluster showing no significant motif. We found no over-represented motif in the top cluster of the enriched HCPBMC as well as CDGUT repertoires (Supplementary Figures S5–8.

We further sought to explore the contribution of public clonotypes in the immune response to gluten. Of the gluten-induced DA clonotypes in each individual as determined by approach one, 10% to 15% were also present in our set of public clonotypes, accounting for an average of approximately 40% and 20% of the cells (reads) in the enriched DA clones of the CDPBMC and CDGUT respectively (Supplementary Table S2 and Figure S9). This estimates the amount of public clonotypes involved in gluten response in each individual, and does not necessarily reflect consistency in response behavior of the clonotypes across patients since most of these clonotypes are not detected from the analysis using approach two. Interestingly, compared to the private DA clonotypes of each patient, higher rate of convergent selection (multiple nucleotide clonotypes coding for identical amino acid clonotype) appears to be a feature of these ‘public subset’ of the DA clonotypes with an increasing trend during gluten exposure (Supplementary Figure S10).

## Discussion

Over the last few years, the T-cell repertoire of various autoimmune diseases has been analyzed using non-biased targeted deep sequencing approaches. In the present study, we have utilized T-cell repertoire deep sequencing and investigated the immune repertoire in gluten exposed CD patients. We profiled and characterized global immune repertoire features in peripheral blood in patients following a short 3-day gluten exposure, and in the gut during active CD. We also identified and characterized clonotypes that showed clonal expansion (or contraction) during gluten exposure.

In our study, the immune repertoire during gluten exposure was generally diverse in both PBMC and gut. We did not detect an alteration of the high TCR diversity observed in CD patient PBMC repertoires. Gluten-specific peripheral CD4+ IFNg+ TCR repertoires were also similar in diversity to the total unsorted patient PBMC repertoires, suggesting high poly-clonality in the CD4+ T-cell response specific to gluten. Hardy *et al*. have reported such diversity at the TCR level in a smaller repertoire of CD4+ IFNg+ T-cells specific to the dominant HLA-DQ2.5-glia-α1a/α2 epitope in both children and adult CD patients, with adult patients not showing any more narrowing of the repertoire specific to the epitope compared to that of children despite more exposure to gluten^18^. The high TCR diversity is also consistent with the cross-reactivity of the gluten-specific T-cells, in which T-cells bearing diverse TCRs are directed to specific immuno-dominant gluten peptides and their homologs^5,18^.

In active CD patient gut biopsies, there was an increase in diversity (by an average of 6%) compared to the treated repertoires after one year, confirming reports that have found higher repertoire diversity in active CD patient biopsies^22^. The increased diversity in the gut reflects the massive increase in mucosal infiltrating T-cells in active CD. It was probably possible to detect this change in the gut due to the relatively stable nature of the gut repertoires, in addition to the gut being the main site of inflammation and/or the longer gluten exposure (Supplementary Figure S11). In general, the immune response associated with gluten exposure appears to involve a diverse set of T-cell clonotypes directed to gluten, and other possible unidentified antigens involved in CD pathology, such as yet to be identified self-antigens and commensal flora, the latter potentially affected by gluten influence on microbiota. Chemokines secreted by the inflammatory cells in active CD could also attract “by-stander” T-cells of many other specificities unrelated to gluten to the site of inflammation.

Gluten exposure led to substantial perturbation of the V-gene usage in both the PBMC and gut repertoires in each patient, mainly due to proliferative changes of the available unique clonotypes. We observed significant over-usage of genes in various TRBV gene families in one or more patients resulting from clonal expansion. We also observed significant alterations in previously reported TRBV genes that are preferentially selected for specific immuno-dominant gliadin epitopes^7,10,11,16^, such as TRBV07-02 (over-used in all patient PBMC repertoires and two gut repertoires) and TRBV04-02 (over-used in four gut repertoires and two PBMC repertoires). Similar analyses of the unique clonotypes suggested that patient repertoires maintained a V-gene usage profile that remained more or less stable before and after gluten exposure, particularly in the gut, likely an indication of clonal persistence in the available immune repertoire.

Inter-individual repertoire overlap was significantly increased among patients during gluten exposure, particularly in the gut, suggesting that the immune response to gluten is characterized by a public component. The evidence for the increased repertoire overlap was even more pronounced when we combined patient gut and PBMC repertoires and compared them to their treated counterparts. While semi-public response to specific immuno-dominant gluten epitopes have been previously reported^7,11^, our data shows that increased amino acid repertoire sharing among CD patients can be detected from the total repertoire (across tissues, and after both a short gluten challenge or long-term gluten exposure) and is likely a major feature of the immune response in CD.

We identified hundreds of differentially abundant clonotypes with significantly altered prevalence during gluten exposure from total repertoires of each individual, both in patients and healthy controls. DeWitt *et al*.^23^ showed that DA clonotypes identified in such manner, i.e. by comparing multiple total repertoires of an individual, captured approximately 60% of known antigen-specific CD8+ cells, whose response magnitude is much higher compared to CD4+ T-cells, in a model of acute viral infection. Since our repertoire data contain response signals coming from both CD8 and CD4 T-cell populations, our estimates are only a rough approximation, possibly an upper bound, of the frequency of gluten-induced T-cells. From these results, we observed that the immune reaction to gluten involved a large private component; public clonotypes constituted between 10–15% of the differentially abundant clonotypes and had an increased rate of convergent selection during gluten exposure. The role and significance of the gluten-induced public clonotypes compared to those that are private in CD pathogenesis is unclear. Public clonotypes are generally less complex and more abundant^24^; TCR sequences that are more likely to be produced in the thymus also pass thymic selection more often^25^, indicating that positive selection of some abundant public clonotypes to the periphery may have a crucial immunological importance. Public clonotypes in mice have been shown to frequently harbor autoimmune specificities^26,27^. They have been shown to have capacity for cross-reactivity, as well as specificity to self and foreign antigens^28^. Self-reactive T-cells have also been associated with more efficient response to foreign antigens in terms of response rates, clonal expansion size and sensitivity to inflammatory signals^29^. Overall, while public clonotypes are likely crucial for efficient immune response, dysregulation of their functions may lead to aberrant self-immunity.

By examining changes in the prevalence of a selected set of public clonotypes, we identified enriched public clonotypes across CD patients in PBMC and gut repertoires. Only one clonotype was shared between the public enriched DA clonotypes of PBMC and gut, possibly due to the differences in the duration of gluten exposure or in the abundance of various T-cell subsets in each compartment; of note, CD patient gut repertoires are dominated by cytotoxic gluten-independent intestinal intraepithelial lymphocytes (IELs) during active disease^30^. In patient PBMCs, the amino acid pattern ASS[LF]R[SW][TD][DT][TE][QA][YF] with conserved usage of the arginine at the fifth position, generalizing previously reported motif ASSxRxTDTQY, was over-represented in the most proliferative cluster of enriched clonotypes, which also contained clonotypes that were exactly identical to the previously reported gluten specific T-cell clones^10^. The arginine residue in gluten reactive clonotypes has been shown to be very crucial in the immune response^9,11,21^. The dominance of such arginine bearing clonotypes is reduced among the public enriched clonotypes of the gut, possibly suggesting that the public clonotypes specific to the highly immunogenic epitopes play an important role mainly in the early response to gluten exposure. This observation of increased arginine bearing clonotypes following gluten exposure is also detectable in total patient PBMC repertoires but not in total gut repertoires, particularly during active disease (Supplementary Figure S12). With prolonged exposure to gluten, at least in the gut, the prevalence of arginine bearing gluten reactive clonotypes may wane, with patient gut repertoires becoming increasingly more diverse and dominated by infiltrating T-cells with clonotypes directed to other antigens. It is possible that arginine bearing gluten reactive public clonotypes may commit to long term-memory more often, as they likely have strong functional avidity to gluten epitopes evidenced by their strong enrichment^31,32^. Once gluten tolerance is broken, these T-cells could be among those that trigger the early adaptive response to gluten upon re-exposure.

This study used small number of samples; the PBMC and gut samples were also not from the same patients. Larger number of samples would have allowed a more representative comparison of repertoires, while matched samples would have provided the possibility to track gluten reactive clonotypes in both compartments. In our experimental setup, we mainly wanted to explore the impact of gluten exposure to the total repertoire in-vivo, which also reflects possible reaction driven by T-cell subsets other than T-helper cells (such as regulatory T-cells, and CD8+ T-cells for which the antigen specificity is unknown), although we did not further study their phenotypes. Thus, the gluten-induced clonotypes we found were not necessarily specific to gluten, but could have been due to secondary effects of its ingestion. Therefore, it would be very informative to study in future the complete profile of the interesting public clonotypes identified, such as their full TCRαβ pairing and gene expression patterns using for instance single cell analysis methods. In spite of these limitations, we were able to identify major immunological signatures associated with gluten exposure, identify gluten-induced clonotypes from total patient repertoires, and estimate contributions of private and public clonotypes to the gluten-induced immune response in patients.

In summary, gluten exposure appears to elicit diverse reaction at the TCR level particularly in the gut, and in PBMC as evidenced by the high TCR diversity observed in the gluten specific PBMC CD4+ IFNg+ repertoires. Increased repertoire overlap was seen in unrelated patients, and the immune response in general was characterized by a large private and an important public component. Deeper level of repertoire sequencing with large number of samples could enable identification and tracking of disease associated public clonotypes or immunological signatures, with potential applications such as early diagnosis, disease and treatment monitoring, and patient stratification based on factors such as patient HLA-types and clinical symptoms.

## Methods

### Subjects and study design

The study participants who provided blood or intestinal biopsy material are presented in Table 1. All subjects were positive for the high-risk CD-associated susceptibility alleles HLA-DQA1*05 and HLA-DQB1*02 encoding HLA-DQ2.5.

In CD subjects, the diagnostic criteria were duodenal villous atrophy accompanied by elevated serum anti-tissue transglutaminase IgA. For the first two experiments, gluten challenges, blood collections, isolation of PBMC and overnight IFN-γ ELISpot were performed according to Anderson *et al*.^4^ and Tye-din *et al*.^5^. CD subjects on long-term GFD consumed one of wheat bread (4 slices, equivalent to approximately 10 g/d gluten), barley flakes (120 g/d, equivalent to approximately 8 g/d hordein), or rye bread (equivalent to approximately 8 g/d secalin). In addition, two healthy subjects who had excluded food with gluten for 4 weeks completed the same 3-day wheat bread challenge. PBMCs were isolated from blood collected immediately before commencing gluten challenge (day 0) and 3 days after completing gluten challenge (day 6). PBMCs were either cryopreserved or used to measure responses to gluten-derived peptides stimulatory for CD4+ T cells in overnight IFN-γ ELISpot assays. In the first experiment, DNA was extracted from frozen PBMC stocks collected before and after wheat gluten challenge from 4 CD donors with positive IFN-γ ELISpot responses to gluten peptides, and 2 healthy donors, for bulk TCR analysis. In the second experiment, PBMC collected from 8 CD donors 6 days after commencing gluten challenge with wheat (n = 3), barley (n = 2), or rye (n = 3) were thawed, and incubated with a mixture of three immunodominant gluten peptides (for antigen presentation by mainly B-cells) to identify CD4+ T cells secreting IFN-γ (see below for details). TCR analysis was performed on CD4+ IFNg+ cells sorted by flow cytometry. In the third experiment, TCR analysis was performed on DNA isolated from fresh 2nd part duodenal biopsies from 5 additional CD patients at the time of diagnosis during active disease and again after one year GFD treatment.

### Ethics

The study design and recruiting of patients were approved by the Ethics Committees of the Pirkanmaa Hospital District, Finland. All subjects gave written informed consent. Relevant guidelines and regulations were followed when performing the experiments.

### Isolation of gluten-specific IFNg-producing T cells

The cryo-preserved PBMC samples were thawed by resuspension into complete RPMI, and re-stimulated, after a resting period of 60 min at 37°C, with a pool of 3 immunodominant gluten peptides (LQPFPQPELPYPQPQ “DQ2.5-glia-α1a & α2”, QPFPQPEQPFPWQP “DQ2.5-glia-ω1 & ω2” and PEQPIPEQPQPYPQQ “DQ2.5-hor-3”)^5,33^, each in a final concentration of 100 microgram/ml. After 16 h, cells were resuspended in PBS, and incubated with IFNg catch reagent, followed by staining with PE anti-IFNg detection antibody (IFNg secretion assay kit; Miltenyi Biotech) and FITC anti-CD4 antibody (clone OKT4; Biolegend), according to manufacturer’s instructions. IFNg secreting CD4+ T cells were separated by FACS sorting, using a BD Facs Aria II instrument. Cells were also stained for gut-homing marker CCR9 in 6 of the 8 samples, which showed that the selected CD4+ IFNg+ population was enriched with cells expressing CCR9 compared to CD4+ IFNg− cell fraction (84% vs 40%, Supplementary Figure S13). DNA was extracted from the sorted cell pellets using a DNA/RNA extraction kit (Qiagen).

### DNA extraction and Sequencing

DNA was extracted (AllPrep DNA/RNA Mini Kit, Qiagen) from 2-10 million pelleted and lysed pre- and post-challenge cell samples of 4 wheat challenged and wheat epitope responding patients, as well as from 2 non-responding healthy controls. For gut samples, total DNA was extracted from snap frozen biopsy specimens, obtained at the time of diagnosis of 5 DQ2 + CD patients with untreated, active disease and confirmed mucosal lesions, as well as biopsies of the same 5 patients after 1 year of gluten-free diet (GFD), using QIAamp Mini Kit (Qiagen, Valencia, CA) as described in Wacklin *et al*.^34^.

DNA samples (1ug per sample) were sent to Adaptive Biotechnologies and were deep sequenced for TCRB CDR3 region (Seattle, USA, www.adaptivebiotech.com; www.immunoseq.com) with their ImmunoSeq assay which employs optimized multiplex PCR to target and amplify the TCR CDR3 region and Illumina for sequencing^35^. For each unique TCRB CDR3 sequence in a sample, the nucleotide and predicted amino acid sequence, the re-arranged V D and J genes, and the number of sequencing reads were determined.

### Sequence data and statistical analysis

Productive sequences without frameshift mutations or stop codons, constituting ~ 80% of our repertoires, were used in all subsequent analyses. We employed downsampling, pre-processing, and data normalization procedures appropriate for each analysis as described in the respective sections. All analyses were done in R (https://www.r-project.org/) and graphs were made using R and Prism 6.0 (GraphPad Software, La Jolla California USA, www.graphpad.com).

Repertoire diversity was estimated using the Shannon entropy index normalized by total number of unique amino acid clonotypes, $(-{\sum }_{{\rm{i}}}\ast {{\rm{C}}}_{{\rm{i}}}\ast {\mathrm{log2}(C}_{{\rm{i}}}))/(\mathrm{log2}(N))$ where ${C}_{i}$ is the relative frequency of unique clonotype $i$ in the repertoire, and $N$ is the total number of unique amino acid clonotypes. A normalized entropy value of 1 reflects high diversity while 0 reflects low diversity. The diversity of every sample was estimated as the median of 100 diversity values calculated from 100 downsampling of each sample to 27077 reads (the size of the second smallest sorted repertoire). Diversity comparison between groups was mainly performed using the non-parametric Wilcoxon Signed Rank Test as assumptions for parametric tests could not be established with the small sample size in this study. But, we also used the parametric paired t-test for some of the analysis since Wilcoxon Signed Rank test has limitations for small sample sizes. For both cases, p-values less than 0.05 were considered significant.

TRBV gene usage was compared between groups of gluten exposed/unexposed repertoires and between the two repertoires of each individual, using information obtained from either unique clonotypes or total reads in repertoires. Comparison between groups was performed using Wilcoxon Signed Rank Test (paired or unpaired as appropriate) with Benjamini–Hochberg (BH) adjusted p-values less than 0.05 considered significant. Comparison between different repertoires sampled from an individual was done using fisher’s exact test with BH adjusted p-values < 0.05 considered significant.

Inter-individual repertoire overlap was calculated for every possible pair of repertoires at each time point using:

1 $${\rm{O}}={\rm{2}}\times [{\rm{c}}{\rm{/}}({\rm{a}}+{\rm{b}})]$$

where O is the estimated inter-individual repertoire overlap between the repertoires, c is the number of identical amino acid clonotypes between the TCR repertoires, a is the number of unique amino acid clonotypes in one of the repertoires, and b is the number of unique amino acid clonotypes in the other repertoire. To avoid possible bias due to differences in sequencing depth, the overlap was estimated as the median of 100 overlap estimates, each calculated after randomly downsampling both repertoires to the total number of sequence reads in the smaller repertoire. The overlap was then compared between groups using Wilcoxon Signed Rank test (paired or unpaired as appropriate) with p-values less than 0.05 considered statistically significant.

Differential abundance (DA) of clonotypes between conditions was performed to identify putative gluten-induced clonotypes whose frequencies differed because of gluten exposure. DA was assessed using two approaches: one was used for identification of DA clonotypes in an individual between repertoires sampled at different time points, and another for identification of public DA clonotypes across individuals by comparing groups of gluten exposed and unexposed repertoires. For approach one, we implemented the fisher’s exact test based-algorithm used for DA analysis by DeWitt *et al*.^23^. In brief, amino acid clonotype abundances were compared between repertoires of an individual sampled at different time points using fisher’s exact test. We adjusted p-values of the shared clonotypes between the two time points using Benjamini–Hochberg (BH) and considered clonotypes with adjusted p-values less than 0.01 differentially abundant. We divided the set of DA clonotypes resulting from this analysis into enriched and de-enriched sets based on their frequencies in the compared repertoires

In approach two, we used the Rank product^36^ combined with sequential forward feature selection (SFS) to identify public DA clonotypes across patients. Briefly, a clonotype abundance (clonotype count frequency) matrix was prepared for public amino acid clonotypes, which we defined as clonotypes seen in at least two different individuals across all repertoire types: namely, CD patient PBMC repertoires (CDPBMC), Healthy control PBMC repertoires (HCPBMC), Sorted sample repertoires (Sorted) and CD patient Gut repertoires (CDGUT). After data normalization, separate two-class rank product analysis was performed using RankProd^37^ for the paired CDPBMC (n = 4), HCPBMC (n = 2), and CDGUT (n = 5) samples. RankProd first ranks clonotypes according to their frequency fold change in the two repertoires sampled from each subject. Next, it computes rank product (RP) for each clonotype across subjects (the geometric mean of the ranks for the clonotype across individuals). It is unlikely that a clonotype has a top ranking fold change consistently in all replicate experiments by chance, i.e. clonotypes with smallest RP, that result from consistent top ranking in replicate experiments, are likely to be enriched or de-enriched clonotypes of interest (depending on the ranking order of the fold changes). The method identifies these differentially abundant features using a permutation test, by estimating the probability of the RP happening by chance for each clonotype. We further filtered the list of clonotypes showing enrichment (i.e., have substantially higher frequency) or de-enrichment (i.e., have substantially lower frequency), with RP p-values less than 0.01, using sequential forward feature selection (from the most significant to the least) to reduce possible false positives. In this step, we selected the first n clonotypes with a leave-one-out cross validation^38^ error less or equal to 0.25 when used in a random forest classification model for prediction of sample’s gluten exposure status. Clonotypes that passed this filter were considered differentially abundant (DA) and were next subjected to cluster analysis. Detailed description of this second approach is available in the supplementary ‘Clonal differential abundance analysis procedure’ section (see also Supplementary Figure S1).

Hierarchical clustering of public DA clonotypes was performed using a modified Levenshtein distance (LD) to estimate pairwise clonotype similarity, by taking into account differences in sequence length and physicochemical properties. The approach combined methods used in previous studies^39–41^. The physicochemical properties were evaluated using hydrophobicity according to the Kyte and Doolittle scale^42^, acidity according to the isoelectric point(PI) and molecular mass(Da) of the amino acids constituting the CDR3 sequences. For each amino acid clonotype, we used the mean property of the amino acids making up the clonotype to represent the clonotype’s overall propensity for that property. Similarity between any two DA clonotypes was then estimated using LD, modified by their difference in length, mean acidity, hydrophobicity, and weight as follows:

2 $$\mathrm{Modified\; LD}=(\mathrm{LD}+{\rm{\triangle }}\mathrm{sequence}\text{\_}\mathrm{lengths}+{\rm{\triangle }}\mathrm{mean}\text{\_}\mathrm{Acidity}+{\rm{\triangle }}\mathrm{mean}\text{\_}\mathrm{Hydrophobicity}+{\rm{\triangle }}\mathrm{mean}\text{\_}\mathrm{Wt})/(\mathrm{Edit}\,{\rm{path}}\,\mathrm{length})$$

The pairwise similarity matrix using this measure was then hierarchically clustered; the dendrogram was cut dynamically^43^ and the resulting clusters were assessed for average fold change values. The cluster containing clonotypes with the highest average fold change was considered the ‘top cluster’. Clonotypes in the top cluster may be considered the most likely proliferative candidates driving the public component of the immune response to gluten. MEME^44^ was used to search for over-represented sequence motifs for clonotypes in each cluster.

### Data availability

The datasets and R scripts used in the current study are available from the corresponding author on reasonable request.

## Electronic supplementary material

Supplementary file

Dataset 1
