# Supplementary material for: Deep sequencing of blood and gut T-cell receptor β-chains reveals gluten-induced immune signatures in celiac disease
Source: Sci Rep. 2017 Dec 21;7:17977. doi: 10.1038/s41598-017-18137-9 (PMC5740085; doi:10.1038/s41598-017-18137-9)
Supplement: Supplementary file 1 — Supplementary file [file 41598_2017_18137_MOESM1_ESM.pdf]

Supplementary Materials

Deep sequencing of blood and gut T-cell receptor β-chains reveals gluten-induced immune signatures in celiac disease

Dawit A. Yohannes[1,2], Tobias L. Freitag[1,3], Andrea de Kauwe[1,2], Katri Kaukinen[4], Kalle Kurppa[5], Pirjo Wacklin[6], Markku Mäki[5], T.Petteri Arstila[1,3], Robert P. Anderson[7], Dario Greco[8], ***Päivi Saavalainen[1,2]

[1] Research Programs Unit, Immunobiology, University of Helsinki, Helsinki, Finland

[2] Department of Medical and Clinical Genetics, University of Helsinki, Helsinki, Finland

[3] Department of Bacteriology and Immunology, University of Helsinki, Helsinki, Finland

[4] Department of Internal Medicine, Tampere University Hospital and Faculty of Medicine and Lifesciences, University of Tampere, Tampere, Finland

[5] Center for Child Health Research, University of Tampere and Tampere University Hospital, Tampere, Finland

[6] Finnish Red Cross Blood Transfusion Service, Helsinki, Finland

[7] Walter and Eliza Hall Institute of Medical Research, Melbourne, Australia. Current address, ImmusanT, Inc., Cambridge MA, USA.

[8] Institute of Biotechnology, University of Helsinki, Helsinki, Finland

Corresponding Author: paivi.saavalainen@helsinki.fi

Supplementary Methods:

Clonal differential abundance analysis procedure (approach two)

In brief, a clonotype abundance (clonotype count frequency) matrix was prepared for public amino acid clonotypes, which we defined as clonotypes seen in at least two different individuals across all repertoire types: namely, CD patient PBMC repertoires (CDPBMC), Healthy control PBMC repertoires (HCPBMC), Sorted sample repertoires (Sorted) and CD patient Gut repertoires (CDGUT). After data normalization, separate two-class rank product analysis was performed using RankProd[1] for the paired CDPBMC (n=4), HCPBMC (n=2), and CDGUT (n=5) samples. In each case, we further filtered the clonotypes showing enrichment (i.e., have substantially higher frequency) or de-enrichment (i.e., have substantially lower frequency) with p-values less than 0.01 using sequential forward feature selection (from the most significant to the least) to reduce possible false positives. In this step, we selected the first n clonotypes with a leave-one-out cross validation[2] error less or equal to 0.25 when used in a random forest classification model for prediction of sample's gluten exposure status. Clonotypes that passed this filter were considered differentially abundant (DA) and were next subjected to cluster analysis. Each step is described below (see also Figure S1).

Clonotype abundance matrix

Unique amino acid (AA) level clonotypes found in all samples and all repertoire types (8 CDPBMC samples, 4 HCPBMC samples, 8 Sorted samples, and 10 GUT samples) were collected into a Clonotype versus Sample data matrix. The total number of unique AA clonotypes was 251259. For each AA clonotype, its clonal abundance (count) in a sample was recorded under the corresponding column for the sample. For AA clonotypes coded by more than one nucleotide clonotype in a sample, the sum of the individual nucleotide level clonotype abundances was used to represent their abundance.

Clone and repertoire selection

The differential abundance (DA) analysis was done on clonotypes that are detected in at least two different individuals across all three repertoire types. There were 11834 such clonotypes (which we refer to as public clonotypes) out of the 251259 sequenced in all three experiments. For DA analysis, we used subsets of the data from the clonotype abundance matrix, where the rows are the public clonotypes and the columns are selected samples of interest for DA analysis depending on the focus of our analysis (For instance, for DA analysis on PBMC repertoires, we select only the CD PBMC samples).

Data transformation and normalization

TCRB Repertoires show high range in clonal abundance values with few clonotypes having high frequency and majority having low frequency, and abundance dependent frequency variation. To improve differential abundance detection and for better interpretation, we add a pseudo-count of one to all clonotype abundances and log-transform the selected data matrix to base 2. This data is then normalized using quantile normalization.

Differential abundance testing

Once we had obtain the normalized data matrix, we perform two class rank product analyses using RankProd[1] with P-values < 0.01 considered significant. The Rank product method identifies differentially abundant features (clonotypes in our data) between groups of samples using a rank product (RP) statistic for fold changes observed in replicate experiments. Briefly, it ranks clonotypes according to their fold change between the two groups/conditions. It computes a rank product for each clonotype (the geometric mean of the

ranks for the clonotype). It is unlikely that a clonotype has a top ranking fold change consistently in replicate experiments by chance. Using a permutation test, the method estimates the probability of the RP happening by chance for each clonotype. Clonotypes with smallest RP, that result from consistent top ranking in replicate experiments unlikely to be obtained by chance, are then more likely to be enriched or deriched clonotypes of interest (depending on the ranking order of the fold changes). We selected clonotypes with p-values < 0.01 for further filtering for differentially abundance (DA).

Differential abundant clonotype filtering

Due to the combined effects of very high noise in the data pertaining to the nature of the data itself, low number of samples and high number of tests, we did not reach significance after multiple testing corrections in the Rank Product analyses. Thus, to help reduce possible false positives in our Rank product results, we performed filtering of the clonotypes with p-values < 0.01 using sequential forward feature selection (from the most significant to the least). In this step, we selected the first n clonotypes with a mean “leave-one-out” cross validation error of ≤ 0.25 when used in a random forest classification model for prediction of repertoire’s gluten exposure status. This was performed for the list of clonotypes that had p-values < 0.01 from the Rank product enrichment analysis, and separately for those with p-values < 0.01 from the derichment analysis. Clonotypes that passed this filtering were considered differentially abundant (DA) as a result of gluten exposure.

Identifying DA clonotypes of interest

Further filtering of the DA clonotypes was done by hierarchical clustering to identify the most interesting DA clonotypes. Hierarchical clustering of differentially abundant (DA) clonotypes was performed using a modified Levenshtein distance (LD) measure to estimate pairwise clonotype similarity by taking into account differences in sequence length and physicochemical properties. The physicochemical properties were evaluated using hydrophobicity according to the Kyte and Doolittle scale[3], acidity according to the isoelectric point(PI) and molecular mass(Da) of the amino acids constituting the clonotype CDR3 sequences. For each amino acid clonotype, we used the mean property (i.e acidity, hydrophobicity or molecular mass) of the amino acids making up the clonotype to represent the clonotype’s overall propensity for that property. Similarity between two clonotype AA sequences was then estimated using LD, modified by their difference in length, mean acidity, hydrophobicity, and weight as follows:

$$Modified\ LD = \frac{LD + \Delta\ \text{sequence\_lengths} + \Delta\ mean\_Acidity + \Delta\ mean\_Hydrophobicity + \Delta\ mean\_Weight}{Edit\ path\ length}$$

The pairwise similarity matrix using this measure was then hierarchically clustered; the dendrogram was cut dynamically[4] and the resulting clusters were assessed for average fold change values. The cluster containing clonotypes with the highest average fold change was considered the ‘top cluster’. Clonotypes in the top cluster may be considered the most likely candidates driving the immune response to gluten. V gene usage was assessed for DA clonotypes. MEME[5] was used to search for over-represented sequence motifs for clonotypes in each cluster.

.

Supplementary Tables:

Table S1. Summary of TCRB CDR3 sequences obtained from samples. The columns represent the three sub-projects (1=whole PBMC TCR repertoires, 2=sorted CD4+IFNg+ cell TCR repertoires, 3=gut biopsy TCR repertoires). Standard deviations are given in parentheses.

	**PBMC (4 CD,2 HC)**		**Sorted CD4+IFNg+ (8 CD)**	**Gut Biopsies (5 CD)**	
AVERAGE	**Pre-challenge (Day 0)**	**Post-challenge (Day 6)**	**Post-challenge (day6,gliadin-peptide restimulated)**	**Active CD**	**GFD (after 1 yr)**
**Total reads**	579883 (151724)	508249 (122919)	114111 (87130)	978273 (194463)	1849847 (996160)
**Unique clones (nt)**	13351 (3000)	16038 (5620)	739 (895)	9561 (2439)	8007 (1880)
**Unique clones(AA)**	13270 (2977)	15933 (5575)	716 (867)	8922 (2253)	7413 (1780)
**Reads/clone (nt)**	45 (16)	33 (7)	191 (105)	108 (32)	226 (107)
**Reads/clone(nt, per 10^6 reads)**	79 (20)	69 (23)	-	113 (41)	133 (32)

Table S2. The number of differentially abundant clonotypes for each subject estimating the number of T cells dynamic upon gluten intake. These represent putative gluten-induced clonotypes when comparison was made between total repertoires of a subject before and after treatment or putative-gluten specific clonotypes when comparison was made between CD4+IFNg+ sorted and unsorted repertoires.

	Number of DA clones in subjects: Putative gluten-induced clonotypes											Number of DA clones in sorted vs total: putative gluten-specific clonotypes			
	CDPBMC				HCPBMC		CDGUT					CD4+INFg+ vs Unsorted(d0)		CD4+INFg+ vs Unsorted(d6)	
	CD005	CD006	CD011	CD039	HC014	HC036	CD1GB	CD2GB	CD3GB	CD4GB	CD5GB	CD005	CD039	CD005	CD039
Enriched	443	452	465	216	241	245	384	1057	1130	592	631	68	235	112	237
In public	48	69	61	35	30	39	50	102	102	67	84	9	36	12	38
In public DA [a]	1	0	3	0	1	2	0	2	1	0	3	1	1	1	0
De-enriched	808	480	226	230	628	157	925	1127	941	984	466	3	53	3	46
In public	76	59	26	28	57	26	73	96	87	101	54	0	7	0	8
In public DA [a]	7	2	5	1	3	3	1	2	4	1	0	0	0	0	3

[a] Results from public DA analysis (approach two) report public clonotypes that show significant differential abundance across individuals on average (not necessarily in every individual) and enables detection of clonotypes that appear in one condition (see Figure 4), whereas DA analysis performed between the two repertoires from each individual imposes the criteria that clonotypes must be shared in the two repertoires (i.e., it misses clonotypes that were absent in either gluten exposed or unexposed conditions). Thus, only a subset of the Public DA clones was available for DA analysis in each individual (since all are not shared between two repertoires of each individual). In addition, some of those that are in both repertoires may not necessarily be differentially abundant in the particular individual.

Supplementary Figures:

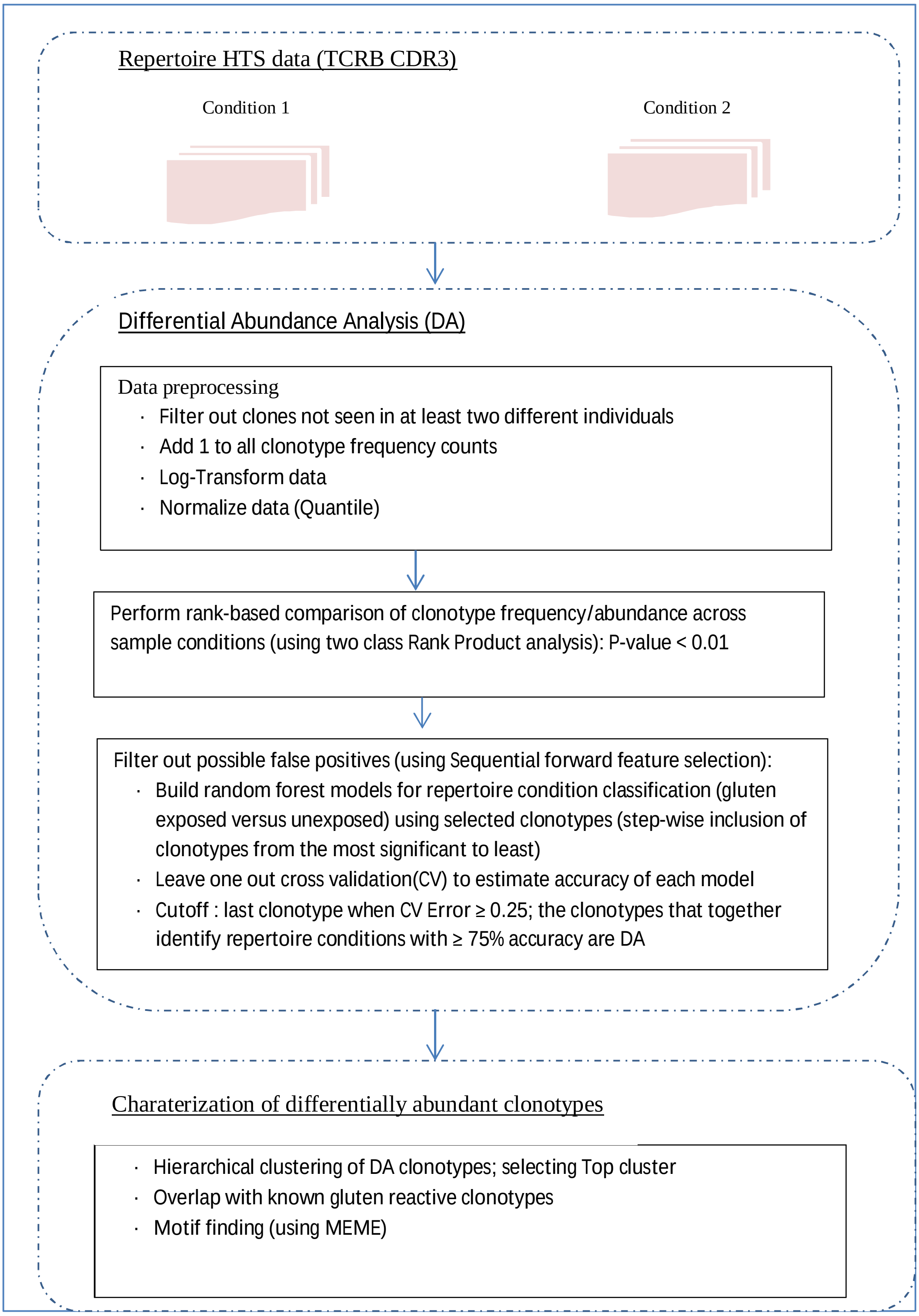

Figure S1. Differential abundance analysis work flow: the work flow for differential abundance analysis of public clonotypes before vs after gluten exposure. The differentially abundant (DA) public clonotypes can further be filtered by selecting the cluster with the highest average fold change after hierarchical clustering of the DA clonotypes

a)

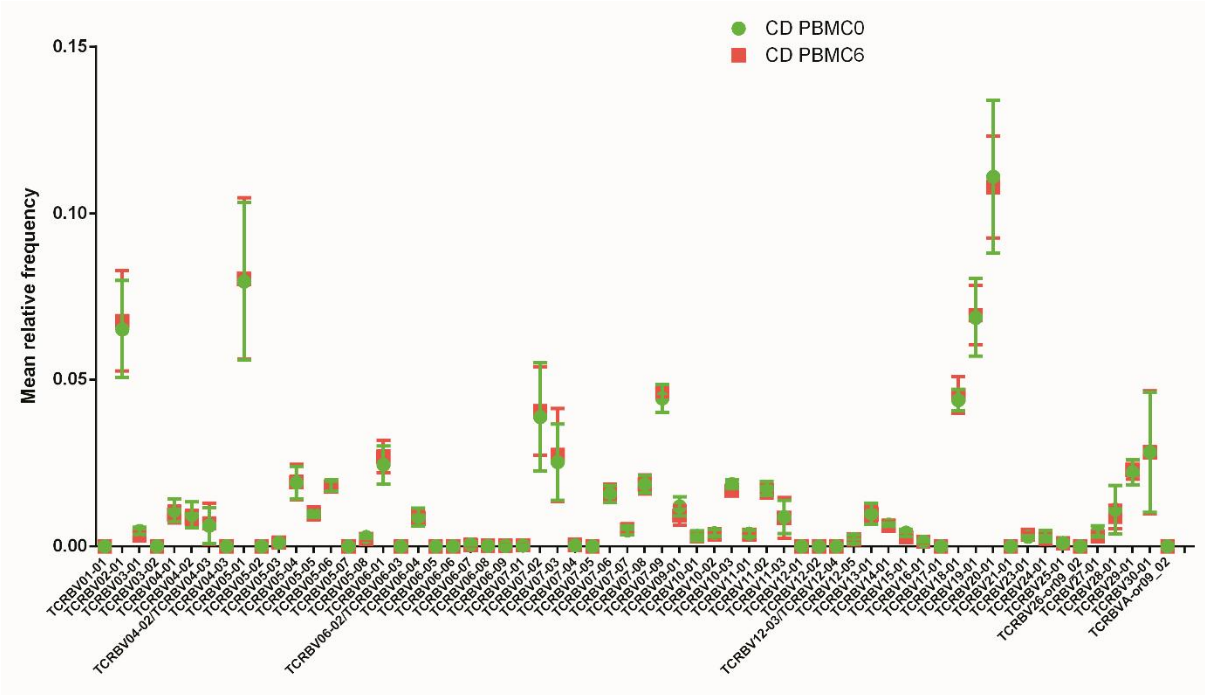
CD PBMC0
CD PBMC6
Mean relative frequency
0.15
0.10
0.05
0.00

b)

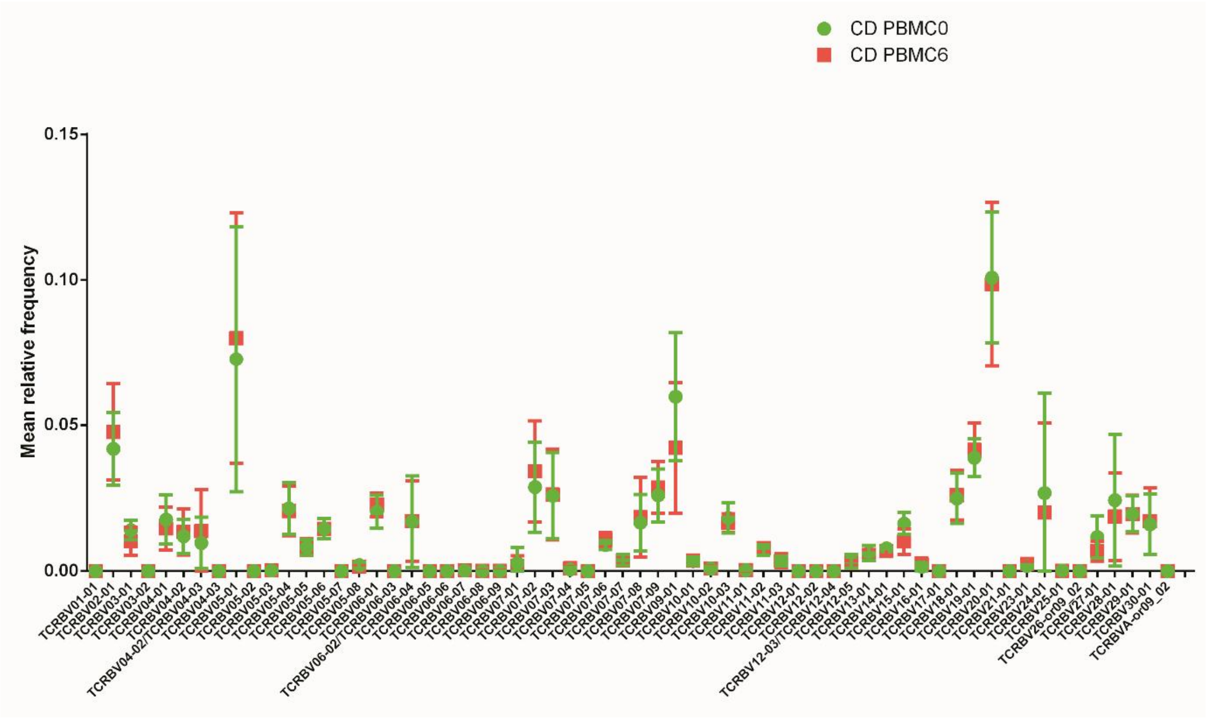
CD PBMC0
CD PBMC6
Mean relative frequency
0.15
0.10
0.05
0.00

c)

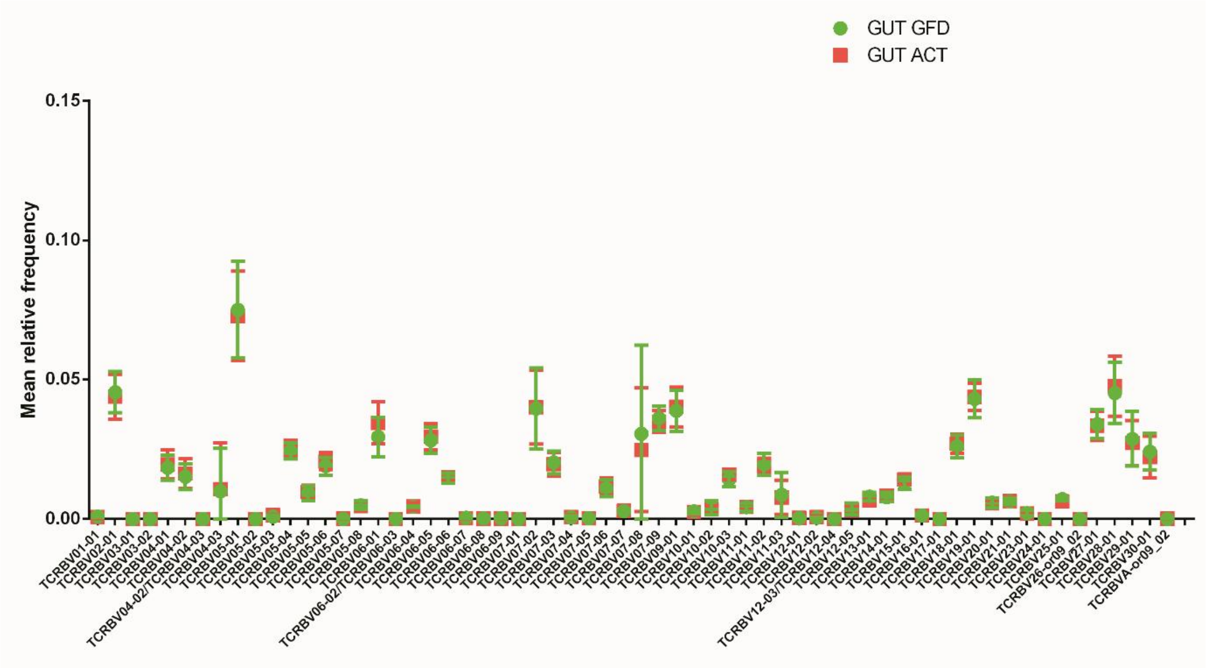

d)

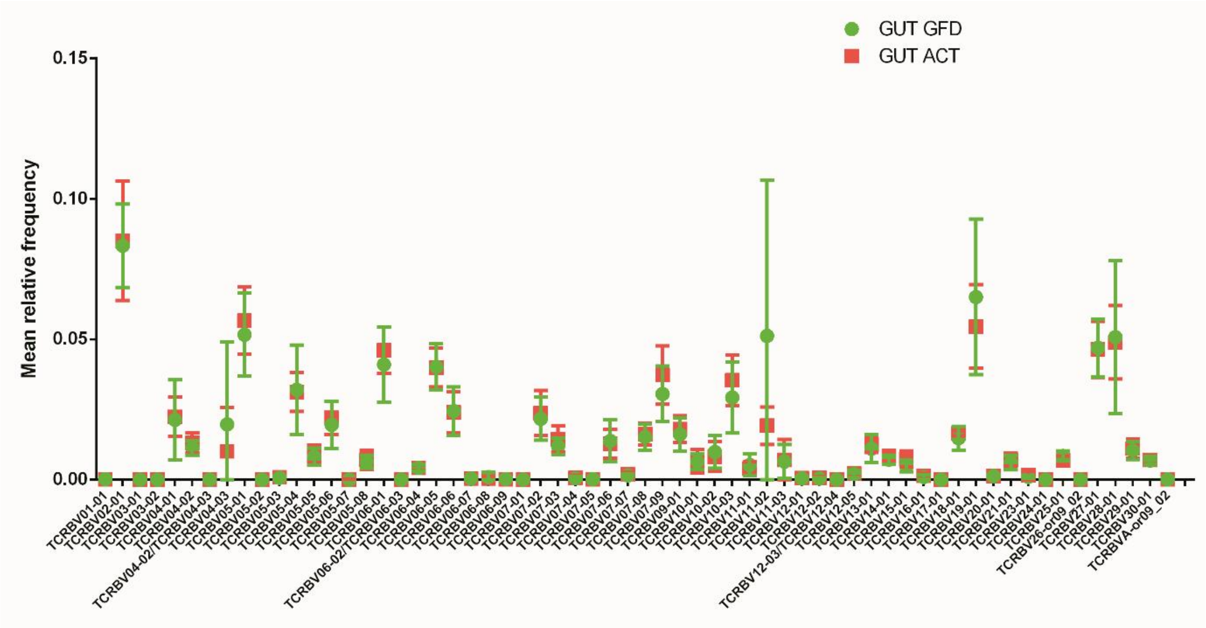

FIGURE S2. Mean TRBV gene usage in treated and untreated CD patient repertoires: (**a**) and (**b**) show mean TRBV usage for CDPBMC unique clonotypes and all sequences respectively. (**c**) and (**d**) show mean usage for CDGUT unique clonotypes and all sequences respectively. Mean ± SD is shown. No significant difference in median TRBV gene usage was detected between treated and untreated repertoires after two-tailed Wilcoxon Signed Rank Test for each gene and multiple test correction using BH.

a)

	CD005	CD006	CD011	CD039
TCRBV03-01	0.01	0.20	0.92	1.00
TCRBV04-01	0.12	0.00	1.00	1.00
TCRBV04-02	0.02	0.04	0.76	1.00
TCRBV05-04	0.26	0.01	0.03	1.00
TCRBV05-08	0.32	0.04	1.00	1.00
TCRBV06-01	1.00	0.71	0.00	1.00
TCRBV07-02	0.03	0.04	0.95	1.00
TCRBV07-03	0.17	0.04	1.00	1.00
TCRBV09-01	0.00	0.00	1.00	1.00
TCRBV15-01	0.13	0.01	0.76	1.00
TCRBV18-01	0.02	0.01	0.22	1.00
TCRBV19-01	0.07	0.05	0.00	1.00
TCRBV20-01	0.02	0.35	0.40	1.00
TCRBV24-01	0.03	0.03	1.00	1.00
TCRBV27-01	0.09	0.00	1.00	1.00
TCRBV28-01	0.43	0.00	0.95	1.00

c)

	CD1G	CD2G	CD3G	CD4G	CD5G
TCRBV06-01	0.01	1.00	0.59	0.85	1.00
TCRBV07-08	0.00	1.00	0.79	1.00	1.00

b)

	CD005	CD006	CD011	CD039
TCRBV02-01	0.00	0.00	0.00	0.53
TCRBV03-01	0.00	0.00	0.00	0.00
TCRBV04-01	1.00	0.00	0.00	0.00
TCRBV04-02	0.37	0.00	0.00	0.00
TCRBV04-02/TCRBV04-03	0.00	0.00	0.00	0.00
TCRBV05-01	0.00	0.00	0.02	0.49
TCRBV05-04	0.32	0.00	0.00	0.31
TCRBV05-05	0.00	0.00	0.00	0.42
TCRBV05-06	0.00	0.00	0.00	0.00
TCRBV05-07	0.36	0.00	0.07	0.14
TCRBV05-08	0.00	0.00	0.12	1.00
TCRBV06-01	0.00	0.67	0.00	0.00
TCRBV06-04	0.00	0.00	0.00	0.00
TCRBV06-07	0.05	0.02	0.00	0.00
TCRBV06-08	0.16	0.00	0.00	0.70
TCRBV06-09	0.00	0.04	0.01	0.00
TCRBV07-01	0.00	0.00	0.00	0.00
TCRBV07-02	0.00	0.00	0.00	0.00
TCRBV07-03	0.00	0.03	0.00	0.01
TCRBV07-04	0.02	0.15	0.00	0.00
TCRBV07-06	0.00	0.00	0.00	0.00
TCRBV07-07	0.00	0.00	0.88	0.31
TCRBV07-08	0.00	0.04	0.83	0.00
TCRBV07-09	1.00	0.00	0.08	0.00
TCRBV09-01	0.00	0.00	0.00	0.00

d)

	CD1G	CD2G	CD3G	CD4G	CD5G
TCRBV01-01	0.02	0.00	0.00	0.00	0.00
TCRBV02-01	0.00	0.00	0.00	0.00	0.00
TCRBV04-01	0.00	0.00	0.00	0.00	0.00
TCRBV04-02	0.00	0.00	0.00	0.00	0.00
TCRBV04-03	0.31	1.00	0.00	0.00	1.00
TCRBV05-01	0.00	0.00	0.00	0.00	0.00
TCRBV05-03	0.00	0.00	0.00	0.00	0.00
TCRBV05-04	0.00	0.00	0.00	0.00	0.00
TCRBV05-05	0.00	0.00	0.00	0.00	0.00
TCRBV05-06	0.00	0.00	0.00	0.00	0.00
TCRBV05-07	1.00	0.00	1.00	0.00	1.00
TCRBV05-08	0.00	0.00	0.00	0.00	0.00
TCRBV06-01	0.00	0.31	0.00	0.00	0.00
TCRBV06-04	0.00	0.00	0.00	0.00	0.00
TCRBV06-05	1.00	0.00	0.00	0.00	0.00
TCRBV06-06	0.00	0.93	0.03	0.00	0.00
TCRBV06-07	0.00	0.00	0.00	0.00	0.00

TCRBV10-01	0.35	0.00	0.00	0.00
TCRBV10-02	0.55	0.36	0.00	0.05
TCRBV10-03	0.00	0.00	0.00	0.00
TCRBV11-01	0.00	0.95	0.02	0.00
TCRBV11-02	0.00	0.75	0.00	0.86
TCRBV11-03	0.01	0.08	0.00	0.00
TCRBV12-05	0.00	0.00	0.00	0.39
TCRBV13-01	0.00	0.00	0.00	0.00
TCRBV14-01	0.00	0.00	0.00	0.12
TCRBV15-01	0.00	0.00	0.00	0.00
TCRBV16-01	0.00	0.00	0.00	0.00
TCRBV18-01	0.00	0.00	0.70	0.13
TCRBV19-01	0.00	0.25	0.00	0.00
TCRBV20-01	0.00	0.00	0.00	0.00
TCRBV23-01	0.00	0.00	0.00	0.70
TCRBV24-01	0.00	0.00	0.00	0.00
TCRBV25-01	0.00	0.00	0.00	0.00
TCRBV27-01	0.00	0.00	0.00	0.00
TCRBV28-01	0.00	0.00	0.00	1.00
TCRBV29-01	0.00	0.00	0.32	0.00
TCRBV30-01	0.00	0.00	1.00	0.00

TCRBV06-08	0.00	0.00	0.00	0.00	0.00
TCRBV06-09	0.00	0.04	0.00	0.00	0.00
TCRBV07-02	0.00	0.00	0.00	0.00	0.00
TCRBV07-03	0.00	0.00	0.00	0.00	0.00
TCRBV07-04	0.00	0.63	0.77	0.00	0.00
TCRBV07-05	0.00	0.00	0.00	0.00	0.00
TCRBV07-06	0.00	0.00	0.00	0.00	0.00
TCRBV07-07	0.00	0.00	0.00	0.00	0.00
TCRBV07-08	0.00	0.00	0.00	0.00	0.00
TCRBV07-09	0.23	0.00	0.01	0.00	0.00
TCRBV09-01	0.00	1.00	0.00	0.00	0.00
TCRBV10-01	0.00	0.00	0.00	0.00	0.00
TCRBV10-02	0.00	0.01	0.00	0.06	0.00
TCRBV10-03	0.00	0.00	0.00	0.00	0.00
TCRBV11-01	0.00	0.27	0.00	0.00	0.00
TCRBV11-02	0.09	0.00	0.00	0.00	0.00
TCRBV11-03	0.00	0.00	0.07	0.00	0.00
TCRBV12-01	0.03	0.00	0.00	0.28	0.12
TCRBV12-02	0.00	0.00	0.00	0.00	0.00
TCRBV12-05	0.00	0.00	0.00	0.00	0.10
TCRBV13-01	0.00	0.00	0.00	0.00	0.00
TCRBV14-01	0.00	0.04	0.00	0.33	0.00
TCRBV15-01	0.00	0.00	0.00	0.00	0.00
TCRBV16-01	0.00	0.59	0.00	1.00	0.00
TCRBV17-01	1.00	1.00	1.00	0.00	1.00
TCRBV18-01	0.00	0.00	0.00	0.00	0.00
TCRBV19-01	0.00	0.00	0.00	0.00	0.00
TCRBV20-01	0.00	0.01	0.00	0.00	0.00
TCRBV21-01	0.00	0.00	0.00	0.00	0.00
TCRBV23-01	0.00	0.20	0.00	0.00	0.00
TCRBV25-01	0.0	0.0	0.0	0.0	0.7

	0	0	0	0	1
TCRBV27-01	0.0<br>0	0.0<br>0	0.0<br>0	0.0<br>0	0.0<br>0
TCRBV28-01	0.0<br>0	0.0<br>0	0.0<br>0	0.0<br>0	0.0<br>0
TCRBV29-01	0.0<br>0	0.0<br>4	0.0<br>0	0.0<br>0	0.0<br>0
TCRBV30-01	0.0<br>0	0.0<br>1	0.0<br>0	0.1<br>8	0.0<br>9
TCRBVA-or09_02	0.0<br>0	1.0<br>0	0.1<br>0	1.0<br>0	0.0<br>0

e)

	Pre-challenge		Post-challenge	
	CD005d0W Vs CD005Wst	CD039d0W Vs CD039Wst	CD005d6W Vs CD005Wst	CD039d6W Vs CD039Wst
TCRBV02-01	0.00	0.00	0.00	0.00
TCRBV03-01	0.00	0.00	0.00	0.00
TCRBV04-01	1.00	0.00	1.00	0.09
TCRBV04-02	0.00	0.00	0.00	0.00
TCRBV04-02/TCRBV04-03	1.00	0.00	0.00	0.57
TCRBV05-01	0.00	0.00	0.00	0.00
TCRBV05-03	0.00	0.00	0.00	0.00
TCRBV05-04	0.00	0.00	0.00	0.00
TCRBV05-05	0.00	0.00	0.00	0.00
TCRBV05-06	0.00	0.02	0.00	1.00
TCRBV05-08	0.00	0.35	0.06	1.00
TCRBV06-01	0.00	0.00	0.00	1.00
TCRBV06-04	0.00	0.00	0.00	0.00
TCRBV06-07	0.00	0.00	0.00	1.00
TCRBV06-08	1.00	0.02	0.09	1.00
TCRBV06-09	1.00	0.00	0.01	0.00
TCRBV07-01	0.00	0.00	0.00	1.00
TCRBV07-02	0.00	0.00	0.00	0.00
TCRBV07-03	0.00	0.00	0.00	0.94
TCRBV07-04	0.00	0.00	0.00	0.00
TCRBV07-06	0.00	0.00	0.00	0.00
TCRBV07-07	0.00	0.00	0.00	0.00
TCRBV07-08	0.00	0.00	0.00	0.00
TCRBV07-09	0.01	0.00	0.01	0.00
TCRBV09-01	0.00	0.00	0.00	0.00
TCRBV10-01	0.00	0.00	0.00	1.00
TCRBV10-02	0.00	0.00	0.00	0.00
TCRBV10-03	0.00	0.00	0.00	0.00
TCRBV11-01	0.00	0.00	0.00	1.00
TCRBV11-02	0.00	0.00	0.00	0.00
TCRBV11-03	0.00	0.00	0.00	0.57
TCRBV12-05	0.00	1.00	0.00	1.00
TCRBV13-01	0.00	0.00	0.00	0.00
TCRBV14-01	0.00	0.26	0.00	1.00
TCRBV15-01	0.00	0.00	0.00	0.00
TCRBV16-01	0.00	0.00	0.00	0.00

f)

	HC014	HC036
TCRBV03-01	0.00	0.61
TCRBV04-01	0.00	0.12
TCRBV06-04	0.00	1.00
TCRBV07-07	0.98	0.01
TCRBV07-08	0.97	0.50
TCRBV07-09	0.36	0.00
TCRBV09-01	0.00	0.00
TCRBV11-02	0.02	0.76
TCRBV15-01	0.00	0.09
TCRBV18-01	0.00	1.00
TCRBV19-01	0.01	0.32
TCRBV24-01	0.02	0.01
TCRBV27-01	0.00	0.09
TCRBV28-01	0.00	0.00

	HC014	HC036
TCRBV02-01	0.00	0.00
TCRBV03-01	0.00	0.00
TCRBV04-01	0.00	0.00
TCRBV04-02	0.00	0.00
TCRBV04-02/TCRBV04-03	0.00	1.00
TCRBV05-01	0.00	0.00
TCRBV05-03	0.00	0.00
TCRBV05-04	0.00	0.00
TCRBV05-05	0.01	0.00
TCRBV05-06	0.00	0.00
TCRBV05-07	1.00	0.00
TCRBV05-08	0.00	0.02
TCRBV06-01	0.00	0.03
TCRBV06-04	0.00	0.03
TCRBV06-07	0.78	0.00
TCRBV06-08	0.04	0.00
TCRBV07-01	0.17	0.00

TCRBV18-01	0.00	0.00	0.00	0.00
TCRBV19-01	0.00	0.00	0.00	1.00
TCRBV20-01	0.00	0.00	0.00	0.00
TCRBV23-01	0.00	0.00	0.00	0.00
TCRBV24-01	0.00	0.00	0.00	0.00
TCRBV25-01	0.05	0.00	0.00	0.57
TCRBV27-01	0.00	0.00	0.00	0.00
TCRBV28-01	0.00	0.00	0.00	0.00
TCRBV29-01	0.00	0.00	0.00	0.00
TCRBV30-01	0.00	0.00	0.31	0.00

TCRBV07-02	0.00	0.09
TCRBV07-03	0.00	0.00
TCRBV07-04	0.00	0.00
TCRBV07-06	0.00	0.11
TCRBV07-07	0.00	0.00
TCRBV07-08	0.00	0.00
TCRBV07-09	0.00	0.00
TCRBV09-01	0.00	0.00
TCRBV10-01	0.05	0.00
TCRBV10-02	0.00	0.00
TCRBV11-01	1.00	0.00
TCRBV11-02	0.00	0.91
TCRBV11-03	0.00	0.01
TCRBV12-05	0.00	0.00
TCRBV13-01	0.00	0.03
TCRBV14-01	0.00	0.21
TCRBV15-01	0.00	0.24
TCRBV16-01	0.00	0.00
TCRBV18-01	0.00	1.00
TCRBV19-01	0.00	0.00
TCRBV20-01	0.00	0.00
TCRBV23-01	1.00	0.00
TCRBV24-01	0.00	0.00
TCRBV25-01	0.00	0.00
TCRBV27-01	0.00	0.00
TCRBV28-01	0.00	0.00
TCRBV29-01	0.00	0.00
TCRBV30-01	0.00	0.66

FIGURE S3. TRBV gene usage in treated versus untreated repertoires for each subject: heat maps show differentially used V genes in repertoires in at least one patient (fisher's exact test, BH adjusted p-values < 0.05), numbers are p-values, green p-values showed odds ratio > 1 (over-representation) and reds OR < 1 (under-representation) during gluten exposure. (**a**) and (**b**) show the result for patient PBMC repertoires when applied to Unique clonotypes and all sequences respectively. (**c**) and (**d**) for patient GUT repertoires when applied to unique clonotypes and all sequences respectively. (**e**) Shows the result of the analysis on all sequences for CD4+ INFg+ vs matched unsorted repertoires (**f**) for analysis on unique clonotypes and all sequences of the two healthy DQ+ control repertoires. Sample names with “G”, gut samples; HC, healthy controls; d0, day 0; d6, day 6; W, wheat challenged PBMC sample; Wst, wheat challenged gluten peptide in-vitro stimulated sorted PBMC sample.

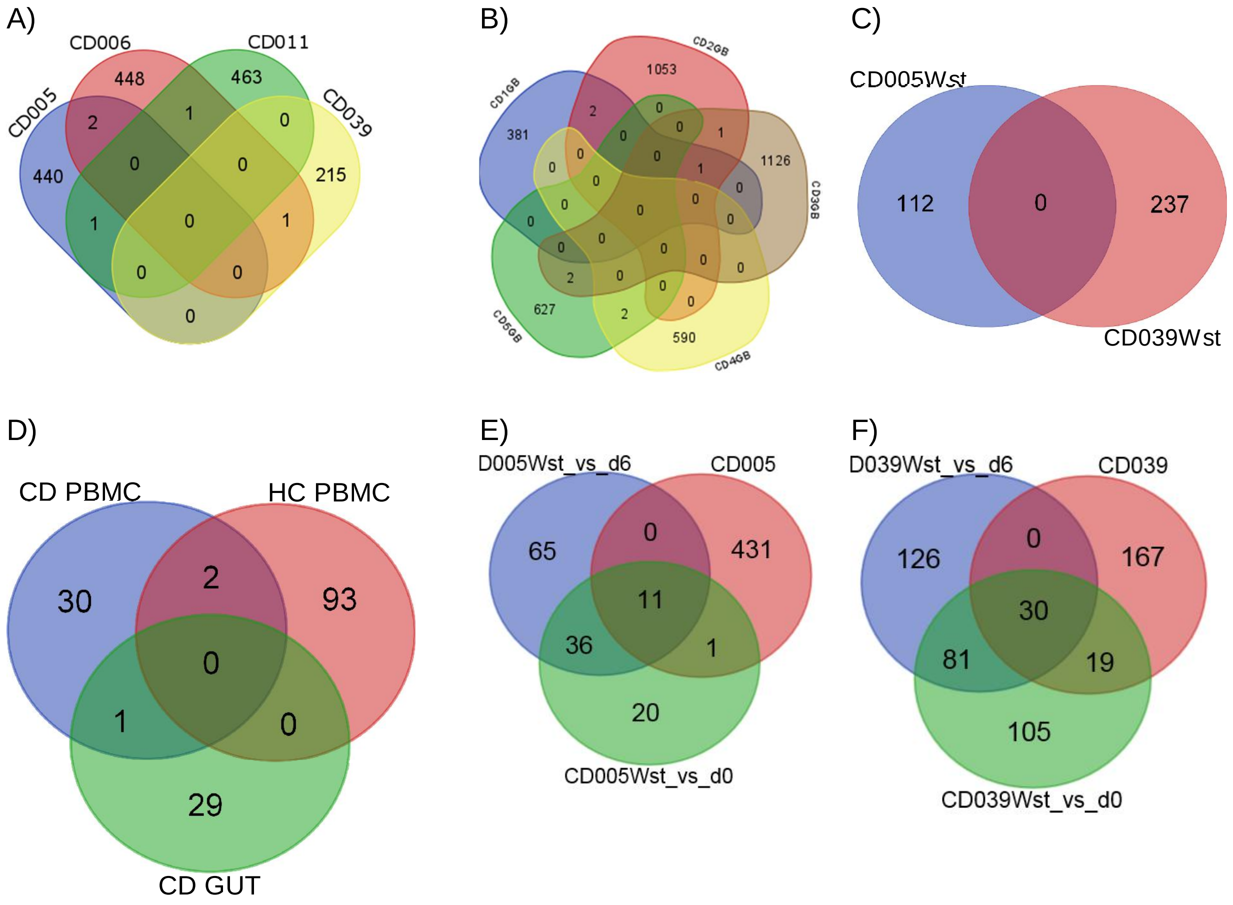

FIGURE S4. Overlap between DA clonotypes in each individual: the number of overlapping enriched clonotypes between patients was low in A) CDPBMC repertoires B) CDGUT repertoires. C) CD005 and CD039 did not share any of their significantly enriched gluten-specific clones in their sorted CD4+IFNg+ repertoires. D) There was also low overlap between public enriched clonotypes detected in patient PBMC and GUT. E & F) There was low overlap of DA clones detected from DA analysis of unsorted repertoires (post-gluten challenge vs pre-challenge), and those detected from sorted vs unsorted repertoire comparison (sorted vs post-challenge, sorted vs pre-challenge) for subjects CD005 and CD039.

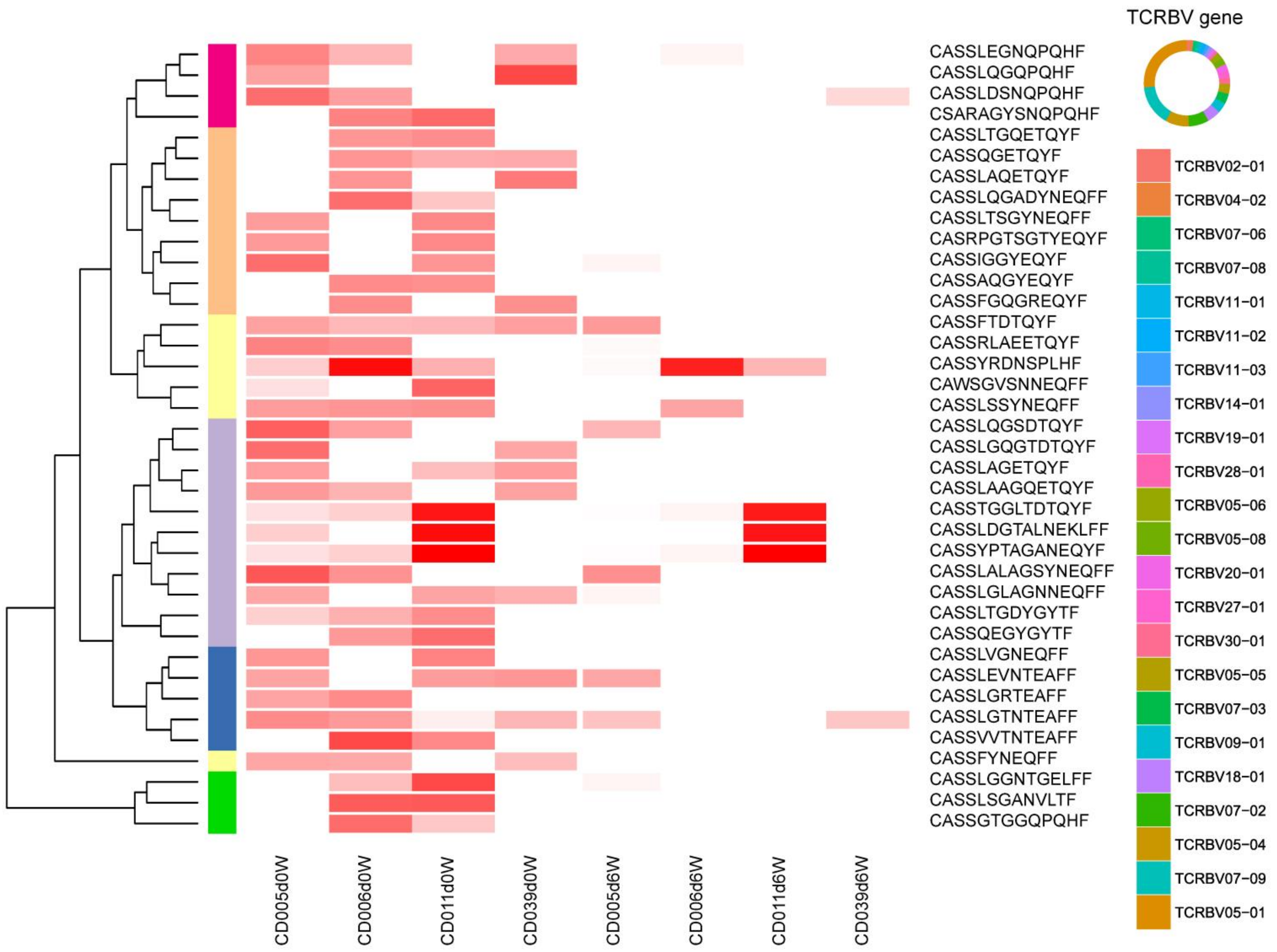

FIGURE S5. Public differentially de-enriched CD PBMC clonotypes: Red color intensity indicates prevalence level; clonotypes are hierarchically clustered using a modified Levenshtein distance that took physicochemical properties into account. The cluster indicated with the green bar has the highest average fold change decrease during gluten exposure. The donut plot on the right shows the TCRBV genes used by all DA clonotypes in CD patient pre-challenge from the least to the most used. Sample names with “CD”, CD patient; d0, day 0; d6 day 6; W, wheat challenged PBMC sample.

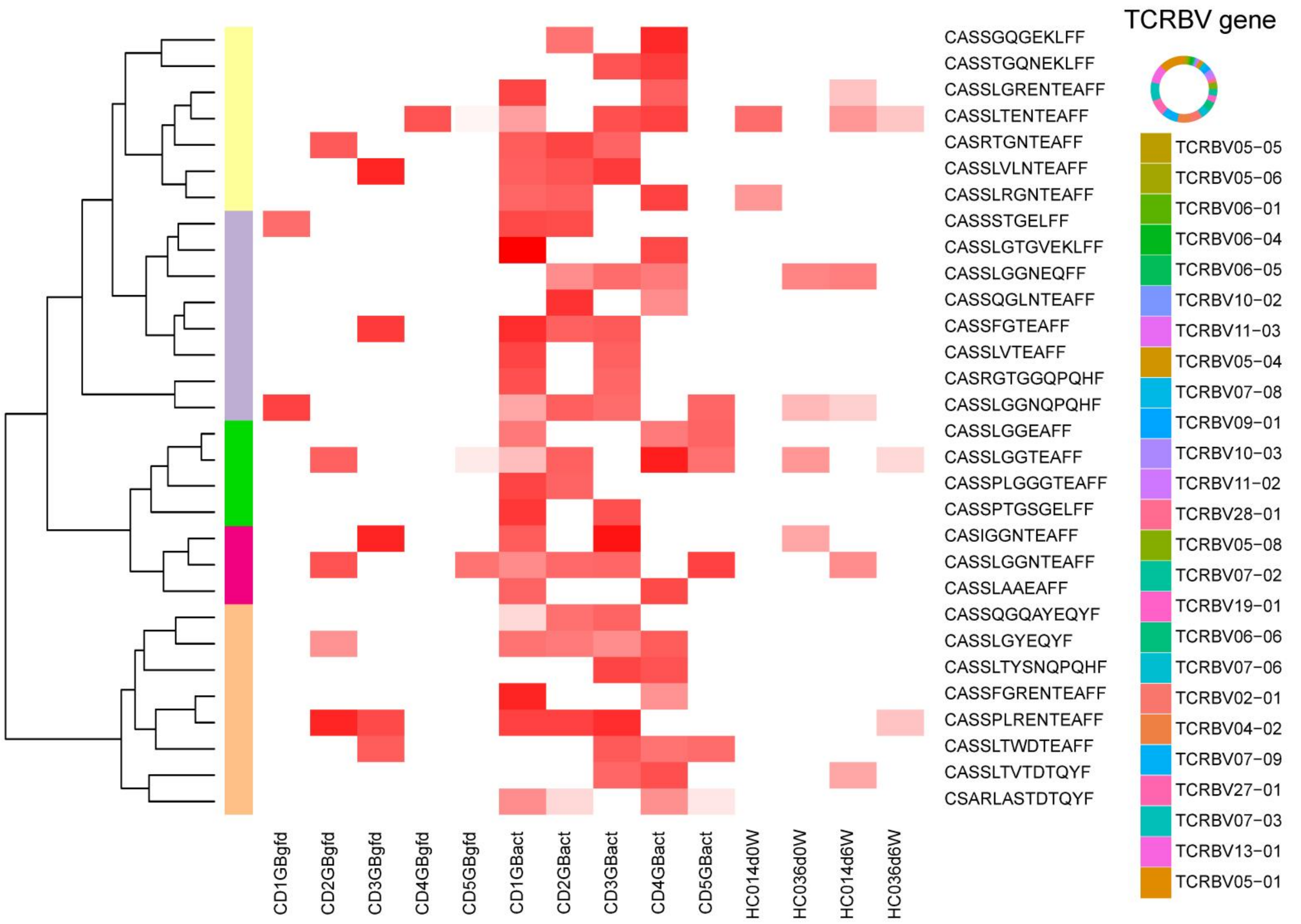

FIGURE S6. Public differentially enriched GUT clonotypes: Red color intensity indicates prevalence level; clonotypes are hierarchically clustered using a modified Levenshtein distance that took physicochemical properties into account. The cluster indicated with the green bar has the highest average fold change increase during gluten exposure. The donut plot on the right shows the TCRBV genes used by all DA clonotypes in CD patient during active disease from the least to the most used. Healthy control samples are included for comparison. Sample names with GBgfd, gut biopsy samples after GFD treatment; GBact, gut biopsy samples from active disease; CD, CD patient

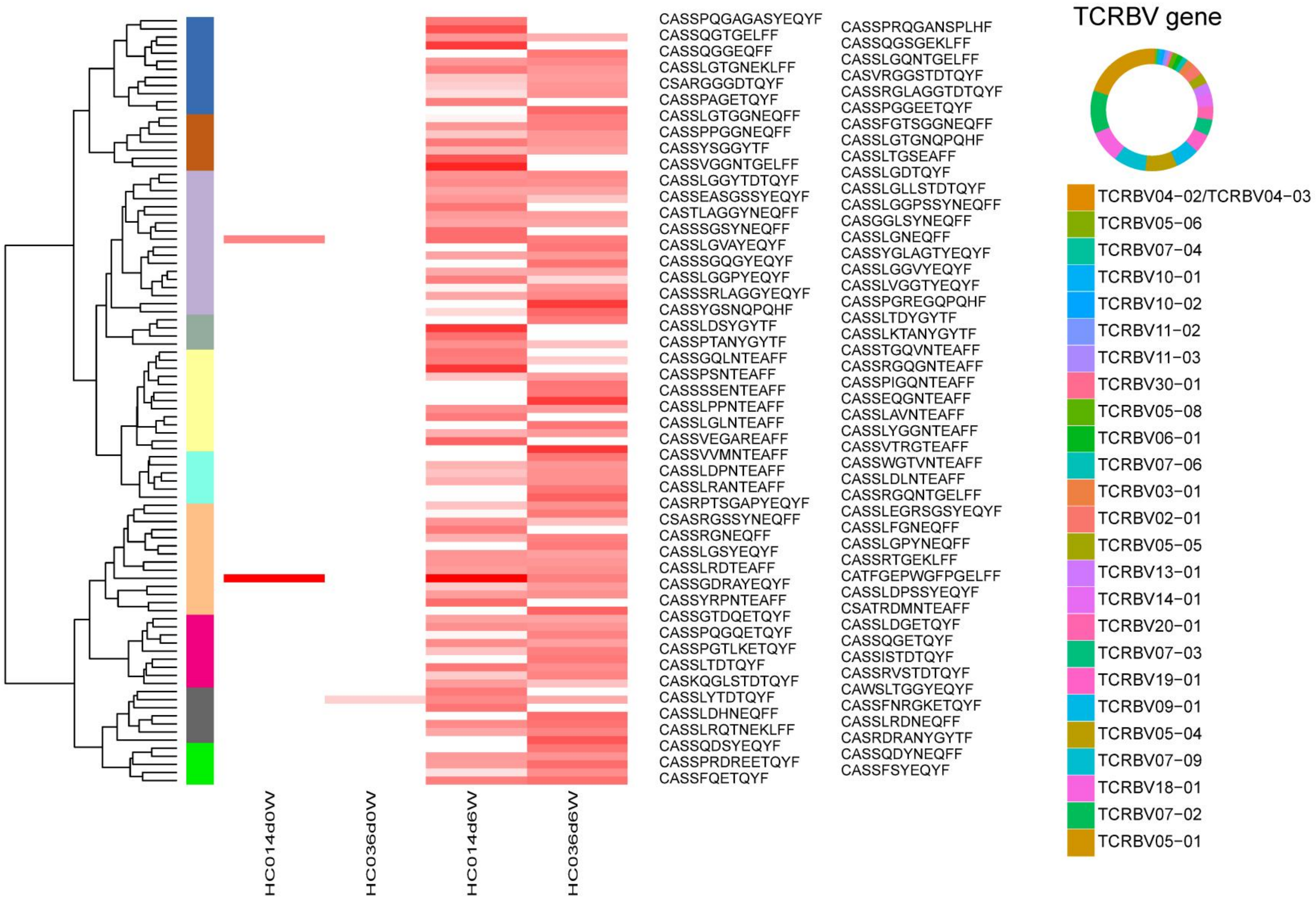

FIGURE S7. Public differentially enriched healthy control PBMC clonotypes: Red color intensity indicates prevalence level; clonotypes are hierarchically clustered using a modified Levenshtein distance that took physicochemical properties into account. The cluster indicated with the green bar has the highest average fold change increase during gluten exposure. The donut plot on the right shows the TCRBV genes used by all DA clonotypes in the two healthy controls following oral gluten challenge. Every other clonotype is shown on the second column for better readability.

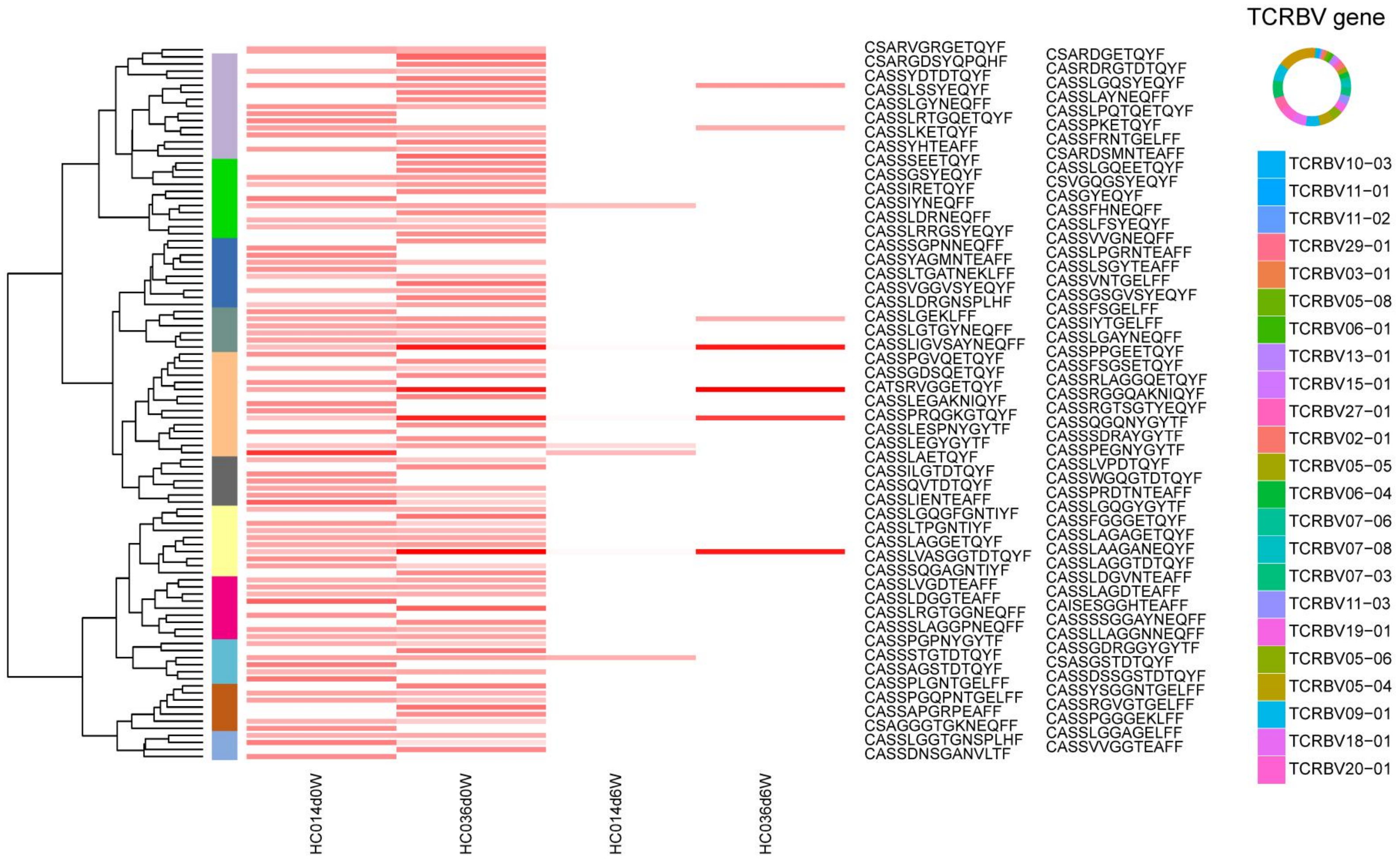

FIGURE S8. Public differentially de-enriched healthy control PBMC clonotypes: Red color intensity indicates prevalence level; clonotypes are hierarchically clustered using a modified Levenshtein distance that took physicochemical properties into account. The cluster indicated with the green bar has the highest average fold change decrease during gluten exposure. The donut plot on the right shows the TCRBV genes used by all DA clonotypes in the two healthy controls following oral pre- gluten challenge. Every other clonotype is shown on the second column for better readability.

A)

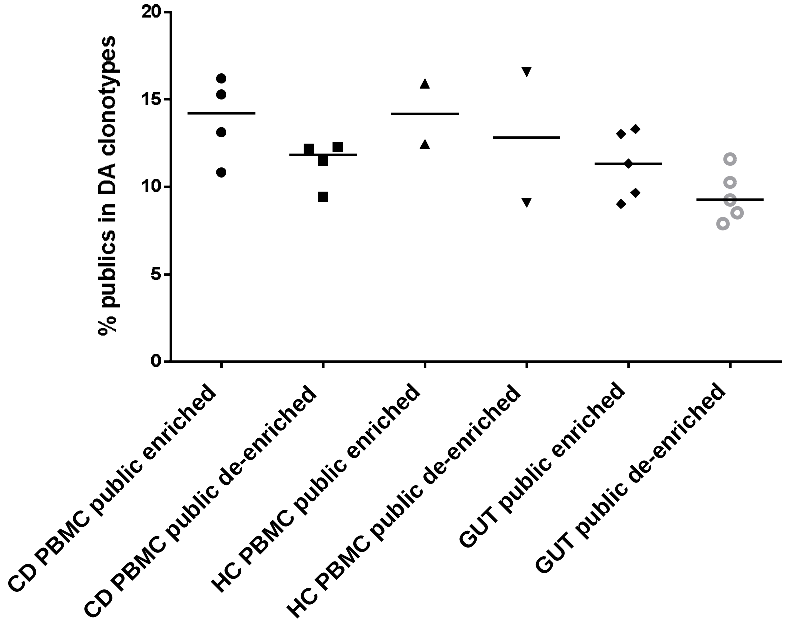

B)

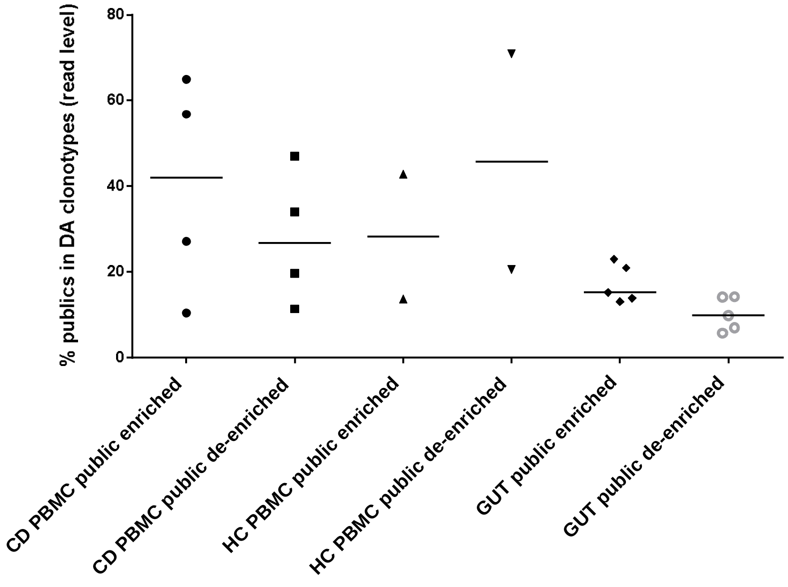

FIGURE S9. Proportion of public clonotypes in DA clonotypes for each subject: We determined percentage of DA clones seen in our public clonotype set for each subject. See Table S1 to find the actual numbers. (A) The average proportion of the public clonotypes was between 10-15% of the total number of DA clonotypes in each subject, (B) making up an average ~40% in PBMC and ~20% in the gut of the cells in the enriched clonotypes of patients. Horizontal bars indicate median.

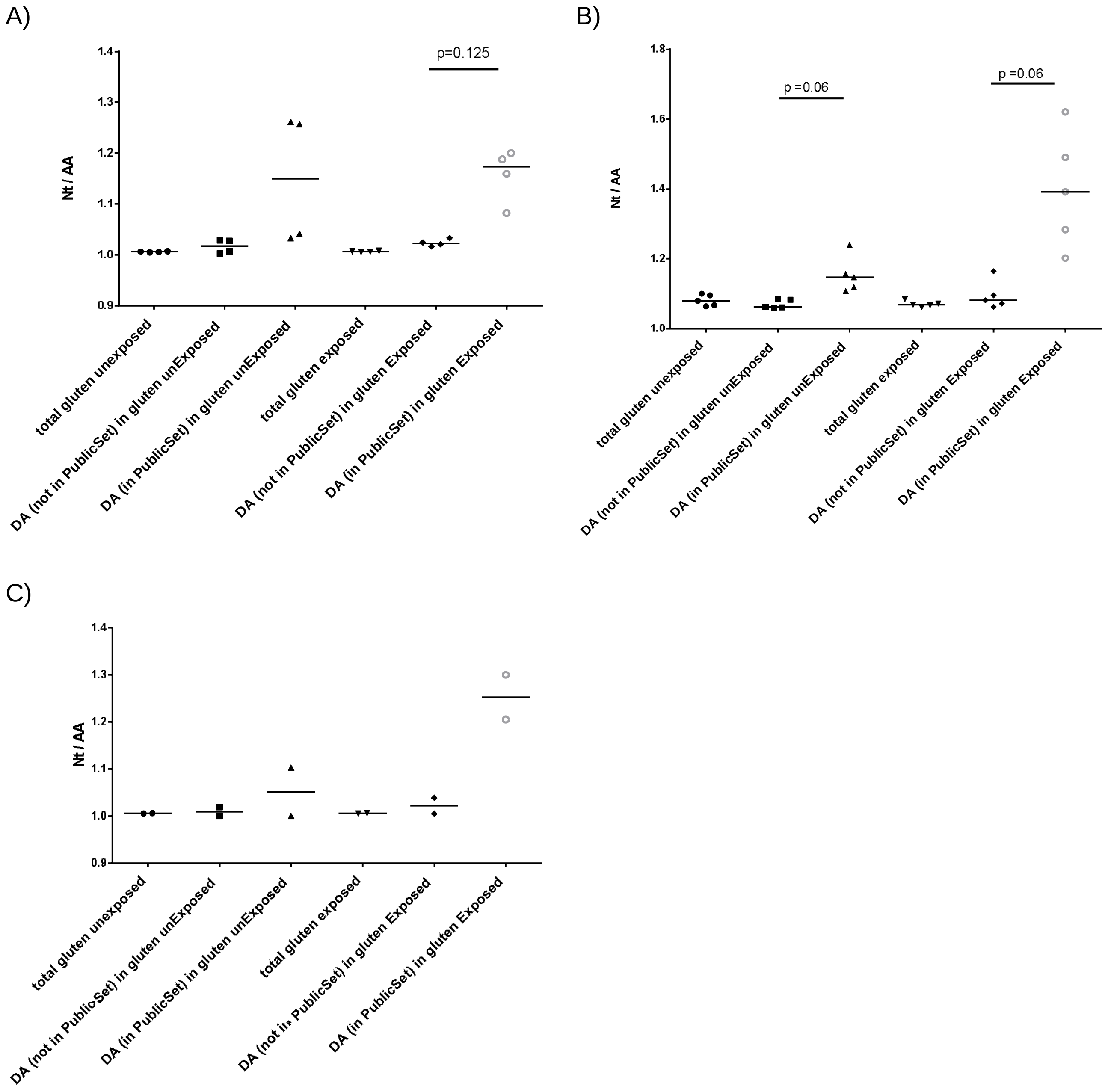

FIGURE S10. Nucleotide to amino acid ratio to compare convergent recombination: convergent recombination is higher in DA clonotypes that are observed in the set of public clones both in gluten unexposed repertoires and in gluten exposed repertoires (with higher increase in the latter). (A) In gluten exposed repertoires, PBMC (two-tailed Wilcoxon Signed Rank Test p = 0.125; paired t-test, p = 0.016). (B) In gluten exposed repertoires, Gut (two-tailed Wilcoxon Signed Rank Test p = 0.0625; paired t-test, p = 0.0066). (C) Healthy controls, the same trend is observed. Overall, the public subset of the DA clonotypes of each subject show higher convergent recombination in gluten unexposed repertoires compared to the private subset, and this trend is increased in gluten exposed repertoires. Horizontal bars indicate median.

.

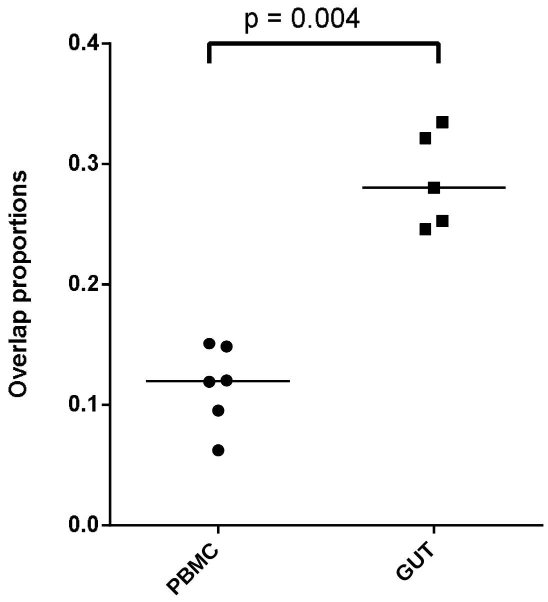

FIGURE S11| Overlap proportions of two repertoires from same individual: Higher overlap in gut repertoires that are sampled 1 year apart compared to PBMC repertoires sampled only 6 days apart (Mann–Whitney U test, two-tailed, p=0.0043). Horizontal bars indicate median. High overlap in the two repertoires sampled from same individual suggests high stability of gut repertoires overtime.

A)

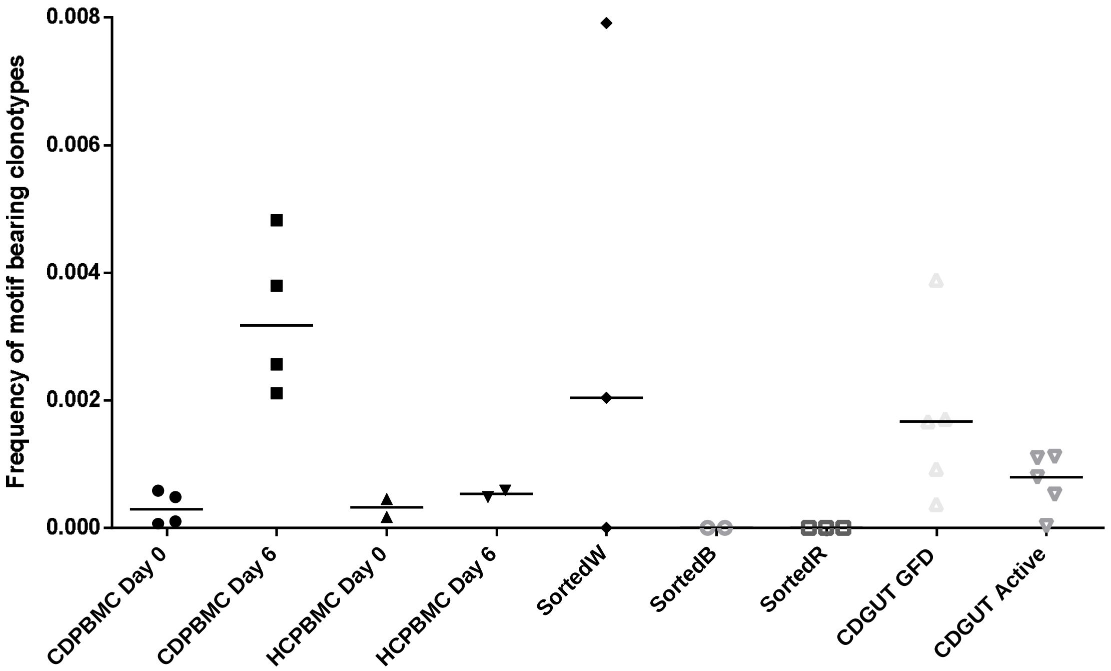

B)

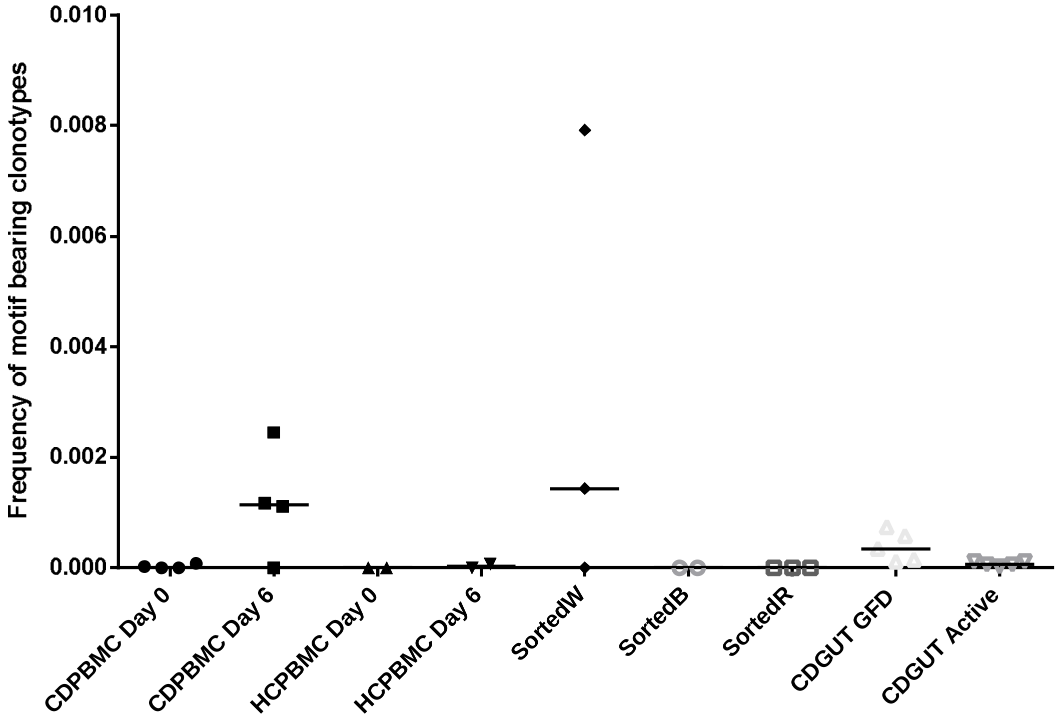

FIGURE S12. Frequency of canonical motif bearing clonotypes in total repertoires: (A) increased frequency of clonotypes bearing the previously identified motif ASSxRxTDTQY (Qiao et al., 2011), (B) and the CDPBMC public enriched top cluster motif from our analysis, ASS[LF]R[SW][TD][DT][TE][QA][YF], is observed in CD patient total PBMC repertoires on day 6. This is observed in the treated gut repertoires instead and not during active disease in the gut. It is also observed in the wheat challenged sorted samples. The plots show values calculated from total reads of the repertoires. Horizontal bars indicate median.

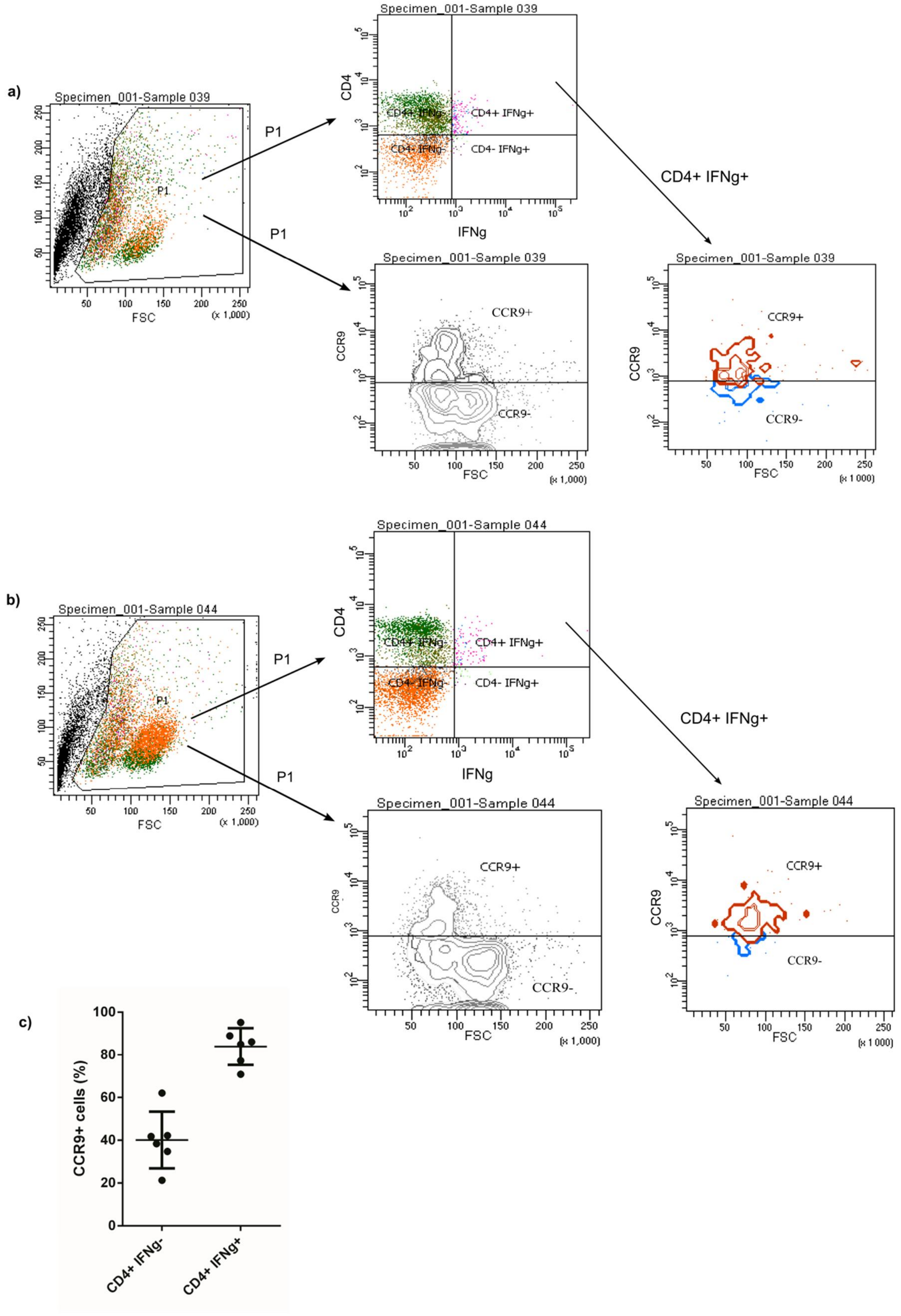

FIGURE S13. Representative plots from FACS sorting experiments (samples CD039 and CD044). Post-oral gluten challenge samples were re-stimulated with three gluten peptides. (a), and (b) We gated on lymphocytes based on forward and side scatter (leftmost plots, P1

gate), and sorted for IFNg secreting CD4+ T cells (upper middle plots, right upper quadrant). CCR9 staining of the gated lymphocytes from P1 demonstrated distinct populations with high and low CCR9 staining intensity (lower middle plots).The gate separating these populations was used to differentiate CCR9+ versus CCR9- CD4+ IFNg+ cells (shown in the rightmost contour plots).(c) The CD4+ IFNg secreting population was enriched in CCR9+ expression, indicating that the majority were gut-homing. Mean ± SD is shown. The total CD4+ IFNg secreting population was submitted to TCR analysis to assess immune features in the gluten peptide-specific repertoire.

REFERENCES of Supplementary material

1. Hong, F. *et al.* RankProd: a bioconductor package for detecting differentially expressed genes in meta-analysis. *Bioinforma. Oxf. Engl.* **22,** 2825–2827 (2006).
2. Arlot, S., Celisse, A. & others. A survey of cross-validation procedures for model selection. *Stat. Surv.* **4,** 40–79 (2010).
3. Kyte, J. & Doolittle, R. F. A simple method for displaying the hydropathic character of a protein. *J. Mol. Biol.* **157,** 105–132 (1982).
4. Langfelder, P., Zhang, B. & Horvath, S. Defining clusters from a hierarchical cluster tree: the Dynamic Tree Cut package for R. *Bioinforma. Oxf. Engl.* **24,** 719–720 (2008).
5. Bailey, T. L. *et al.* MEME SUITE: tools for motif discovery and searching. *Nucleic Acids Res.* **37,** W202–208 (2009).
